# Ileal bile acid transporter inhibition in *Cyp2c70* KO mice ameliorates cholestatic liver injury

**DOI:** 10.1016/j.jlr.2022.100261

**Published:** 2022-08-05

**Authors:** Jennifer K. Truong, Ashley L. Bennett, Caroline Klindt, Ajay C. Donepudi, Sudarshan R. Malla, Kimberly J. Pachura, Alex Zaufel, Tarek Moustafa, Paul A. Dawson, Saul J. Karpen

**Affiliations:** 1 Department of Pediatrics, Division of Pediatric Gastroenterology, Hepatology and Nutrition, Emory University School of Medicine, Children's Healthcare of Atlanta, Atlanta, Georgia, USA; 2 Division of Gastroenterology and Hepatology, Department of Internal Medicine, Medical University of Graz, Graz, Austria

**Keywords:** Cytochrome P450, cholesterol 7α-hydroxylase, sterol 12α-hydroxylase, hydrophobicity, detergency, enterohepatic circulation, muricholic acids, chenodeoxycholic acid, biliary cell proliferation, BA, bile acid, CA, cholic acid, CDCA, chenodeoxycholic acid, Ck-19, cytokeratin-19, DCA, deoxycholic acid, HI, hydrophobicity index, IBAT, ileal bile acid transporter, IBATi, ileal bile acid transporter inhibitor, KEGG, Kyoto Encyclopedia of Genes and Genomes, LCA, lithocholic acid, MCA, muricholic acid, RBC, red blood cell, TCA, taurocholic acid, TCDCA, taurochenodeoxycholic acid, TDCA, taurodeoxycholic acid, TUDCA, tauroursodeoxycholic acid, UDCA, ursodeoxycholic acid

## Abstract

Cyp2c70 is the liver enzyme in rodents responsible for synthesis of the primary 6-hydroxylated muricholate bile acid (BA) species. *Cyp2c70* KO mice are devoid of protective, hydrophilic muricholic acids, leading to a more human-like BA composition and subsequent cholestatic liver injury. Pharmacological inhibition of the ileal BA transporter (IBAT) has been shown to be therapeutic in cholestatic models. Here, we aimed to determine if IBAT inhibition with SC-435 is protective in *Cyp2c70* KO mice. As compared to WT mice, we found male and female *Cyp2c70* KO mice exhibited increased levels of serum liver injury markers, and our evaluation of liver histology revealed increased hepatic inflammation, macrophage infiltration, and biliary cell proliferation. We demonstrate serum and histologic markers of liver damage were markedly reduced with SC-435 treatment. Additionally, we show hepatic gene expression in pathways related to immune cell activation and inflammation were significantly upregulated in *Cyp2c70* KO mice and reduced to levels indistinguishable from WT with IBAT inhibition. In *Cyp2c70* KO mice, the liver BA content was significantly increased, enriched in chenodeoxycholic acid, and more hydrophobic, exhibiting a hydrophobicity index value and red blood cell lysis properties similar to human liver BAs. Furthermore, we determined IBAT inhibition reduced the total hepatic BA levels but did not affect overall hydrophobicity of the liver BAs. These findings suggest that there may be a threshold in the liver for pathological accretion of hydrophobic BAs and reducing hepatic BA accumulation can be sufficient to alleviate liver injury, independent of BA pool hydrophobicity.

## Graphical abstract

**Figure:**
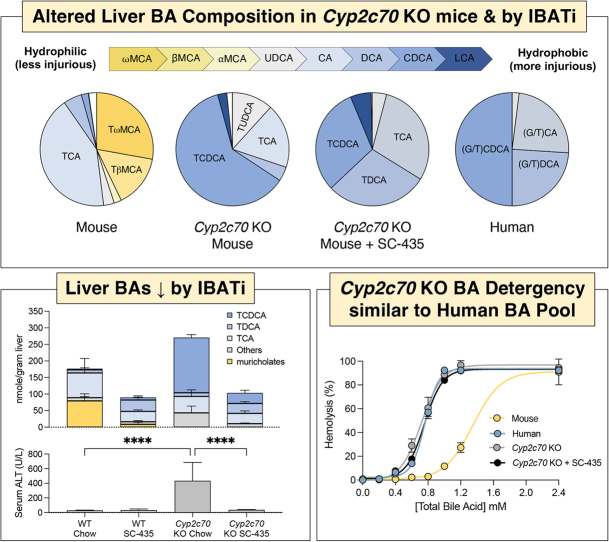

Bile acids (BAs) are synthesized from cholesterol in all vertebrate species, secreted into bile and empty into the intestine, where they play an important role in the digestion and absorption of lipids and fat-soluble vitamins (1). Beyond their role as detergents, BAs also function as signaling molecules and regulate cellular and whole-body metabolism (2). The amphipathic properties of BAs that enable effective micellization and transport of lipids in aqueous environments such as the biliary tract and gut lumen also confer the potential for cytotoxicity when the normal secretion and compartmentalization of BAs in the enterohepatic circulation is impaired (3, 4). Despite being the subject of considerable study (3, 5, 6, 7), the role of BAs and toxic bile in the pathogenesis of human cholestatic liver disease remains a fundamental unresolved question (8, 9, 10). The use of laboratory animal models, particularly the mouse, has played a significant role in developing our understanding of BA homeostasis and mechanisms of BA-mediated injury (8, 9, 10). Importantly, species-specific differences in BA compositions need to be considered when attempting to use mouse models to understand mechanisms of BA-related disease and injuries in humans (11). These differences include a structurally distinct, more hydrophilic BA composition (2) and a ∼7-fold higher BA biosynthesis rate in mice (50 mg/kg/day) than that in humans (7 mg/kg/day) (12). These differences significantly impact the BA’s physicochemical and signaling properties, metabolism, interaction with the microbiome, and potential for cytotoxicity, thereby presenting translational challenges when attempting to model the BA-induced injury in human cholestatic liver disease (11). Particularly important for the mouse-human BA composition comparisons is the abundant hepatic synthesis of 6-hydroxylated primary BA species (muricholates) in mice, 7α-rehydroxylation of conjugated deoxycholic acid (DCA) and lithocholic acid (LCA) in mice but not humans, and the amino acid specificity for N-acyl-amidation (conjugation) of the BA side chain in mice (almost exclusively taurine conjugates) versus humans (∼2.5:1 mixture of glycine to taurine conjugates in bile) (11).

The cytochrome P450, Cyp2c70, was recently identified as the enzyme responsible for muricholate synthesis in mice (13, 14, 15, 16), converting chenodeoxycholic acid (CDCA) (3α,7α-hydroxy) and ursodeoxycholic acid (UDCA) (3α,7β-hydroxy) to α-muricholic acid (MCA) (3α,6β,7α-hydroxy) and β-MCA (3α,6β,7β-hydroxy) (17). As a result of the step catalyzed by Cyp2c70 during primary BA synthesis, hydrophilic and less injurious 6-hydroxylated MCAs constitute almost half of the circulating BA pool in mice. In contrast, MCAs are not present in the BA pool of humans, who lack *Cyp2c70* or a gene that performs a similar enzymatic function. To better model the role of the more hydrophobic and injurious human BA pool in the pathogenesis of disease, several groups have begun characterizing *Cyp2c* locus and *Cyp2c70* KO mice, models with a more human-like BA composition (13, 16, 17, 18). *Cyp2c70* KO mice spontaneously develop neonatal cholestasis, as evidenced by significant increases in serum biomarkers and changes in histology and gene expression characteristic of cholestatic liver injury (14, 17). The liver injury phenotype was quantitatively more severe in female mice, however, male mice also exhibited significant liver damage. The absence of protective MCAs and increased production of CDCA contributes to the increased hydrophobicity and cytotoxicity of the BA pool, resulting in hepatic injury and BA retention. Indeed, treatment of *Cyp2c70* KO mice with the hydrophilic BA UDCA reduced the hydrophobicity of the BA pool and normalized liver histology and hepatic injury markers (18). In the present study, we explored the concept that the BA contribution to liver injury extends beyond their hydrophobicity. Common to the reported *Cyp2c70* KO mouse models generated is an increase in the liver total BA content (13, 17, 18). In the *Mdr2* KO (*Abcb4* KO) model of cholestatic liver injury and sclerosing cholangitis, pharmacological inhibition of the ileal BA transporter (IBAT/ASBT; *Slc10a2*) increases fecal BA elimination, leading to reductions in liver BA concentrations and improvement in biomarkers of hepatocellular and cholestatic damage (8, 19). IBAT inhibitors are being evaluated in clinical trials as an anticholestatic therapy (20) and have demonstrated clinical benefit in children and been approved for the treatment of progressive familial intrahepatic cholestasis and Alagille syndrome (21, 22). In the following study, we tested whether blocking IBAT-mediated return of BAs to the liver in the enterohepatic circulation would be protective in *Cyp2c70* KO mice and investigated the predicted cytotoxicity of the humanized BA pool composition in this model. IBAT inhibition did not change the overall hydrophobicity of the liver-associated BAs but significantly reduced the liver accretion of BAs in *Cyp2c70* KO mice leading to normalization of liver histology, gene expression, and serum biomarkers of liver injury to WT levels.

## Materials and methods

### Materials

The IBAT inhibitor (IBATi) SC-435; (4R,5 R)-5-[4-[4-(1-aza-4-azoniabicyclo[2.2.2]octan-4-yl)butoxy] phenyl]-3,3-dibutyl-7,8-dimethoxy-1,1-dioxo-4,5-dihydro-2H-1λ6-benzothiepin-4-ol was received as a research gift from Shire Pharmaceuticals.

### Animals

All animal experiments were approved by the Institutional Animal Care and Use Committees at Emory University. *Cyp2c70* KO mice (C57BL/6 background) were generated by Gene Edit Biolabs (Atlanta, GA) using CRISPR-Cas9 technology by microinjecting Cas9/gRNA into single-cell stage mouse embryos to produce founder (F0) mice. A strategy targeting exon 2 of *Cypc270* was used and followed the approach described by Honda *et al.* (14). The plasmid DNA J1-3 encoding the gRNA and Cas9 was used for microinjection. The *Cyp2c70* gRNA right target sequence was AGATGATTATTAGTGTA and the left target sequence was CTCTTGTCACTGTTCCA. The resulting F0 mouse encoded a deletion of 1,277 bp that included the entire targeted region encompassing exon 2 (supplemental Fig. S1A). The mice were genotyped by real-time quantitative PCR and DNA sequencing. The primers used to detect the WT *Cyp2c70* allele were forward primer 5′-TCTTCTTGCCTTCAACAGCA-3′ and reverse primer 5′-AACCATTGCACAGAGCACAG-3′ and yielded a product size of 662 bp. To detect the mutant *Cyp2c70* allele encoding the exon 2 deletion, the same forward primer was used with reverse primer 5′-GAAAGCCCATGAGAGAGGAA-3′ and yielded a product size of 350 bp (supplemental Fig. S1B). The F0 mouse was bred to the next generation (F1) and subsequent offspring from F4-F5 were confirmed by genotyping. The male and female WT and *Cyp2c70* KO mice were born at expected Mendelian ratios and used for our in vivo studies. The female breeding mice were fed ad libitum rodent breeder chow (21% of calories as fat; PicoLab Diet 20 No. 5058; PicoLab Cat. No. 0007689) and maintained in cages with standard bedding (one-eighth” Bed-O-Cobbs; Andersons Lab Bedding Products) and pulp cotton fiber nesting material (Nestlets; Anacare). The offspring were weaned at 4 weeks instead of 3 weeks of age since this appeared to improve viability, in agreement with the reported findings for an independent line of *Cyp2c70* KO mice (18). Absence of Cyp2c70 protein in livers of *Cyp2c70* KO mice was confirmed by Western Blot analysis using an antibody raised against amino acids 366–390 (24 amino acids, PRKTTQDVEFRGYHIPKGTSVMAC) (23) (supplemental Fig. S1C). The WT and *Cyp2c70* KO mice were maintained on a C57BL/6 background. The adult mice were maintained on ad libitum rodent chow (13% of calories as fat; PicoLab Rodent Diet 20; LabDiet) and group-housed in ventilated cages (Super Mouse 750 Microisolator System; Lab Products) containing standard bedding at 22°C in the same 12:12h light/dark cycle–controlled room of animal facility to minimize environmental differences. For the study, male and female WT and *Cyp2c70* KO mice were fed chow or chow plus 0.006% (w/w) SC-435, which provided approximately 11 mg/kg/day of the IBATi (24).

### Mouse sample collection and analysis

The mice were fed ad libitum and not fasted prior to blood collection and euthanasia to collect tissues. Mouse blood was collected into MiniCollect Z Serum Separation tubes (Greiner Bio-One #450472) from the submandibular vein. The serum was isolated by centrifugation at 1500 g for 20 min at room temperature. Serum chemistries including alkaline phosphatase, alanine aminotransferase (ALT), aspartate aminotransferase (AST), and BAs were measured at the Emory University Department of Animal Resources Quality Assurance and Diagnostic Laboratory using an Alfa Wassermann Vet Axcel chemistry analyzer. Tissue collection: following euthanasia under isoflurane anesthesia, the mouse livers were perfused with 3 ml of PBS and harvested. After weighing, the liver tissue was subdivided as previously described (25). Lobes 2 and 5 were flash frozen in liquid nitrogen for Western Blot and RNA analysis. Lobe 4 was embedded in optimal cutting temperature compound for frozen section preparation. The remainder of the liver lobes (1, 3, 6, and 7) were used for histological analysis. Mouse spleens were also harvested and weighed.

### Histological analysis and immunohistochemistry

The liver samples were fixed in 10% neutral formalin (Sigma-Aldrich) for 24 h, transferred to 70% ethanol, embedded in paraffin, and processed for sectioning by Children’s Healthcare of Atlanta Pathology Services. Histological sections (5 μm) were cut. Consecutive sections were used for staining with H&E and Sirius Red. For immunohistochemistry, liver section slides were deparaffinized and subjected to antigen retrieval using Rodent Decloaker (Biocare #RD913M). To block endogenous peroxides, the sections were incubated for 15 min in BLOXALL Readymade Solution (Vector Labs #SP6000). The sections were washed with MilliQ water and then incubated in Powerblock solution (Biogenex #HK0855K) for 15 min, followed by incubation with 3% donkey serum diluted in PBS for 1 h at room temperature. The slides were subsequently incubated with primary antibody overnight at 4°C (1:500 Ck-19 Abcam #ab52625, 1:100 F4/80 Cell Signaling #70076). The following day, slides were washed 3 times in PBS with 0.1% Tween 20 for 5 min, incubated in Signal Stain Boost IHC detection (Cell Signaling #8114) for 45 min, and washed. The color was developed using Signal Stain DAB substrate (Cell Signaling #11725) and counterstained with hematoxylin (Sigma #HHS16) for 30 s. The slides were dehydrated with increasing ethanol concentrations (70%–100%) and placed in xylene. The slides were then mounted using Vectamount Permanent Solution (Vector Labs #H5000).

### Image analysis

Immunohistochemistry slides were scanned with the 40X objective using NanoZoomer (Hamamatsu). Quantification of Sirius Red staining and immunohistochemistry for cytokeratin-19 (Ck-19) and F4/80 was performed using the latest QuPath release (version 0.2.3) (26). Briefly, Ck-19 and F4/80 immunohistochemistry slides were scanned (ndpi files) and imported into QuPath. The entire tissue section was selected as a single annotation using the built-in Magic Wand tool. For Ck-19 and F4/80 slides, cell counts were carried out using the positive cell detection tool to identify hematoxylin-stained nuclei and then thresholding for DAB positive-staining cells to determine the number of DAB-positive cells/mm^2^ tissue. Identical settings and thresholds were applied to all slides for a given stain and experiment. Positive Sirius Red staining was quantified using the pixel classification tool with a residual threshold of -0.12. Sirius Red positive tissue was expressed as the percent of pixels above the threshold for the total tissue area examined. All slides for a given experiment were set to specific parameter thresholds with minimal adjustments for staining variation.

### Gene expression measurements

Total RNA was extracted from frozen liver tissue using TRIzol reagent (Invitrogen, Carlsbad, CA) and a RNeasy Mini Kit (Qiagen), and cDNA was generated using a High Capacity cDNA Reverse Transcription Kit (Applied Biosystems). Real-time PCR was performed on a QuantStudio™ 5 Real-Time PCR System (Applied Biosystems) using a SYBR Green qPCR Master Mix (Bimake.com). Quantification of relative gene expression was conducted by calculating fold change relative to cyclophilin D as a reference gene using the ΔΔ C(t) method. The mouse primer sets used are listed in the supplemental methods. For RNA-Seq and gene set enrichment analysis, total RNA was extracted from frozen liver tissue using TRIzol reagent (Invitrogen, Carlsbad, CA). RNA-Seq libraries were prepared by Novogene Co., Ltd and sequenced on an Illumina HiSeq1000 system. Differential expression analysis was performed using the DESeq2 R package of Bioconductor (27). The resulting *P* values were adjusted using the Benjamini-Hochberg procedure to control for the false discovery rate (28). Differentially expressed genes with a log2 fold change > 1.0 and adjusted *P* < 0.05 (multiple testing false discovery rate 5%) were selected for functional annotation (GEO series accession number: GSE183251). Pathway analysis of the RNA-Seq data was performed using MetaCore (GeneGo Inc, Saint Joseph, MI) and the Kyoto Encyclopedia of Genes and Genomes (KEGG) and Database for Annotation, Visualization, and Integrated Discovery (DAVID) (29, 30).

### Protein expression measurements

For Western blot analysis, total protein extracts were prepared from flash frozen liver tissue by homogenization in RIPA buffer (30 mM Hepes pH 7.4, 150 mM NaCl, 1% Nonidet P-40, 0.5% sodium deoxycholate, 0.1% sodium dodecyl sulfate, 5 mM EDTA), supplemented with a combined protease and phosphatase inhibitor cocktail. Proteins were reduced and denatured in Laemmli sample buffer containing fresh DTT, resolved on 4%–12% Bis-Tris gels, transferred to nitrocellulose membranes, immunodetected with antibodies, and imaged using a ChemiDoc system (BioRad). The source of the primary and secondary antibodies used in the study are provided in the Supplemental Information, supplemental Table S2.

### Liver BA determination

The liver BA content was measured using previously described methods (31). Briefly, frozen liver tissue samples (40–60 mg each) were extracted using Folch’s extraction procedure to remove apolar lipids (lower phase) and precipitated proteins (interphase) before the addition of deuterated internal standards (32). Analytes were separated by HPLC using a C18 reversed phase column prior to quantification by mass spectrometry using a combination of deuterium-labeled internal standards and unlabeled standards. The following BAs were measured: cholic acid (CA), UDCA, CDCA, DCA, LCA, and their glycine and taurine conjugates; as well as αMCA, βMCA, γMCA (hyocholic), ωMCA, and their taurine conjugates.

### Red blood cell lysis assay

Whole blood is harvested from an anesthetized adult C57BL/6 mouse by cardiac puncture and collected into a tube with EDTA at a final concentration of 1.8 mg/ml (5 mM). Whole blood is centrifuged at 2500 rpm for 5 min at room temperature. The plasma and buffy coat are aspirated off, and the red blood cell (RBC) pellet is resuspended in buffer A (10 mM CaCl_2_, 150 mM NaCl, 25 mM glucose, 10 mM Tris-HCl, pH 7.4) at a ratio of 10 ml of buffer A per 1 ml of packed RBCs. Calcium at a final concentration of 10 mM was included to enhance the hemolytic activity of the BAs (33). The cells were washed 3 times in buffer A by centrifuging at 2500 g for 5 min. After the final wash, RBCs were resuspended in buffer A (1 ml of packed RBCs is diluted up to a final volume of 40 ml) and kept on ice until ready to use. Taurine- and glycine-conjugated BA sodium salts were dissolved in buffer A and diluted to the desired final BA concentration in a 150 μl total volume and added to round bottom 96-well plates prior to the addition of 142 μl of washed RBC suspension. The mixture was then incubated at 37°C for 30 min. After incubation, the plate is centrifuged at 4500 g for 5 min at room temperature to pellet unlysed cells. A 100 μl aliquot of the supernatant containing the free hemoglobin released from lysed RBCs is transferred to a clear, flat bottom 96-well plate and absorbance is read at 515 nm. Hemolysis for the individual BAs or BA mixtures is reported as percent of the RBC lysis induced by the positive control (0.1% Triton-X 100).

### Cell culture, MTT, and lactate dehydrogenase assays

Alpha mouse liver 12 cells (AML12, ATCC) were used to measure BA toxicity. AML12 cells were cultured at 37°C in 5% CO_2_ and maintained in DMEM (4.5 g/l glucose) supplemented with 10% fetal bovine serum, 1% penicillin/streptomycin, and 20 mM L-glutamine. For the cell viability assays, AML12 cells were plated on collagen I–coated 96-well plates at a density of 10,000 cells/well. The next day, the cells were preincubated in complete medium containing the indicated BAs for 1 h and then treated for 2 h with a final concentration of 1 mg/ml of the MTT reagent (3-(4, 5-dimethylthiazol-2-yl)-2, 5-diphenyltetrazolium-bromide) (Molecular Probes™ M6494). Absorbance of each well was read using a BioTek™ Synergy™ HTX Multi-Mode Microplate (Molecular Devices, Sunnyvale, CA) at a wavelength of 570 nm and the percent cell viability reported was normalized to vehicle control. To measure lactate dehydrogenase release, parallel 96-well plates of AML12 cells were preincubated for 4 h in complete media containing the indicated BAs and then for 30 min with reagent as according to manufacturer’s protocol (BioLegend, catalog #42640). Following the addition of stop solution, absorbance was measured at 490 nm and cytotoxicity was reported relative to internal high and low controls.

### Statistical analyses

For the box and whisker plots, median values (line), interquartile range (boxes), and min to max values (whiskers) are shown. For the liver BA composition analysis and BA toxicity assays, mean value ± SD is shown. The data were evaluated for statistically significant differences using the Mann-Whitney test, the two-tailed Student’s *t* test, ANOVA and Tukey-Kramer honestly significant difference post-hoc test or Sidak’s multiple comparisons test (GraphPad Prism; Mountain View, CA). Differences were considered statistically significant at *P* < 0.05.

## Results

### IBAT inhibition protects against *Cyp2c70* deficiency-associated liver disease

**Fig. 1:**
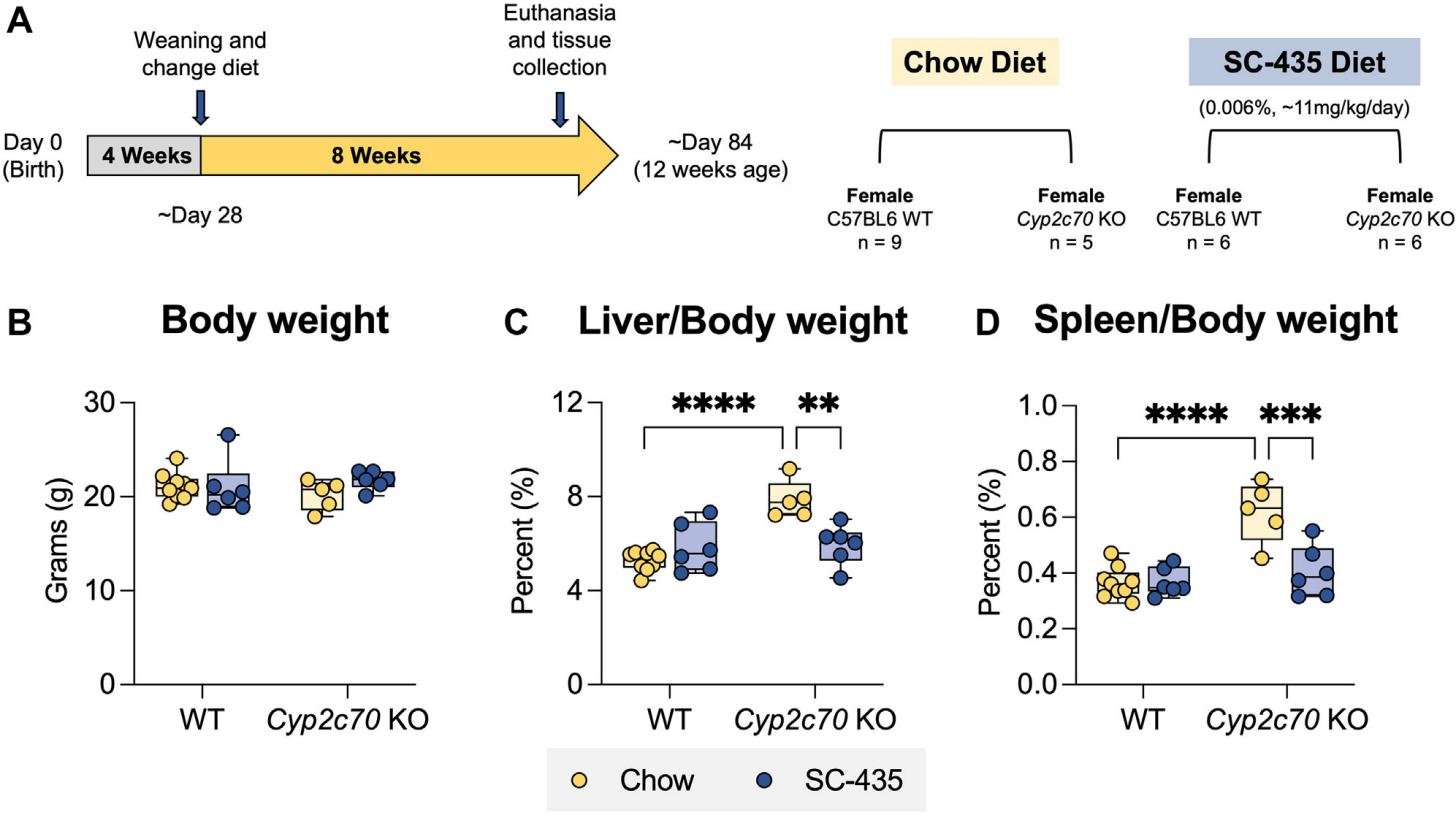
Liver and spleen weights are elevated in female *Cyp2c70* KO mice. A: Experimental scheme. Mice are weaned at 4 weeks and maintained on chow or switched to SC-435 diet until sacrifice and tissue was collected at 12 weeks of age. B: Body weights. C: Liver/Body weights. D: Spleen/Body weights. Asterisks indicate significant differences between groups. Median values (line), interquartile range (boxes), and min to max values are shown (*∗∗P* < 0.01, ∗∗∗*P* < 0.001, ∗∗∗∗*P* < 0.0001); n = 5–9 mice per group.

**Fig. 2:**
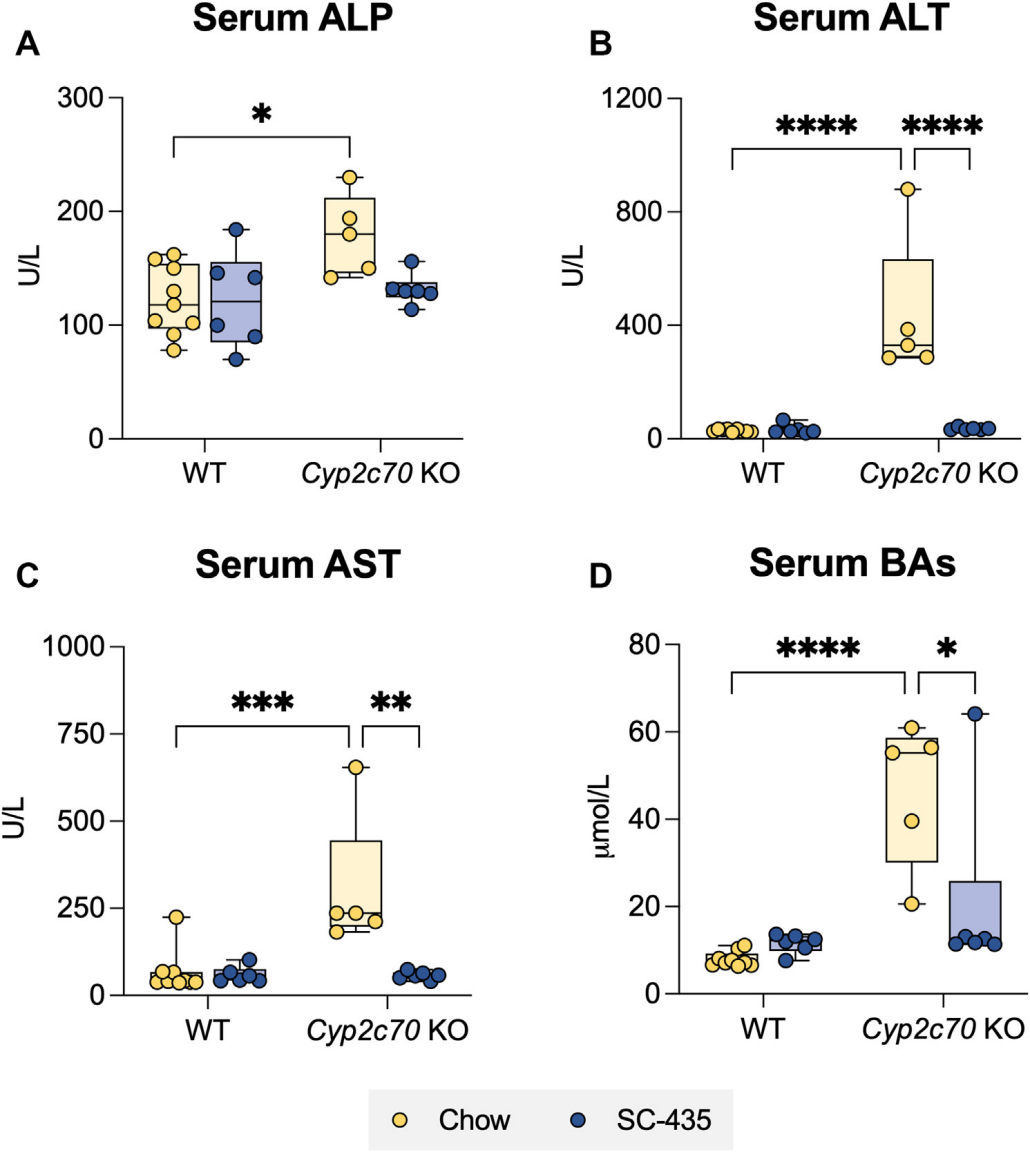
Female *Cyp2c70* KO mice have elevated serum liver injury markers and are protected from cholestatic injury by the IBAT inhibitor SC-435. A: Serum alkaline phosphatase (ALP). B: Serum alanine aminotransferase (ALT). C: Serum aspartate aminotransferase (AST). D: Serum bile acids (BAs). Asterisks indicate significant differences between groups. Median values (*line*), interquartile range (*boxes*), and min to max values are shown (*whiskers*) (*∗P < 0.05, ∗∗P* < 0.01, ∗∗∗*P* < 0.001, ∗∗∗∗*P* < 0.0001); n = 5–9 mice per group. IBAT, ileal BA transporter.

To determine whether interrupting the enterohepatic circulation of BAs with an IBATi is protective against development of the liver injury associated with *Cyp2c70* deficiency, male and female WT and *Cyp2c70* KO mice were fed chow or chow supplemented with 0.006% SC-435 (∼11 mg/kg/day) for 8 weeks as outlined in Figs. 1A and S2A. In agreement with previously reported studies (14, 18), the liver injury phenotype is more pronounced in female *Cyp2c70* KO mice. For clarity, figures in the main text show the results for female mice and findings for male mice are presented in the Supplemental Materials. The body weights of *Cyp2c70* KO mice–fed chow or chow plus IBATi were lower than WT mice at 4 weeks of age (supplemental Fig. S1D), becoming similar to WT mice by 12 weeks for both female (Fig. 1B) and male (supplemental Fig. S2B) mice. As previously reported, the liver to body weight ratio (Figs. 1C and S2C) is significantly elevated in *Cyp2c70* KO mice. In addition, *Cyp2c70* KO mice had a significantly higher spleen to body weight ratio, which suggests the presence of liver injury–associated portal hypertension in these animals (Figs. 1D and S2D) (34). Treatment with IBATi blocked the increase in liver and spleen weights in *Cyp2c70* KO mice. Female and male *Cyp2c70* KO mice maintained on chow diet exhibited strongly elevated serum levels of liver injury markers as compared to WT mice. This included levels of alkaline phosphatase, an indicator of cholestatic liver injury, which were significantly elevated in female (Fig. 2A) and male (supplemental Fig. S3A) *Cyp2c70* KO compared to WT mice of the same age. The same trend was observed for serum levels of the liver cytosolic enzymes ALT (Figs. 2B and S3B) and AST (Figs. 2C and S3C) and for BAs (Figs. 2D and S3D) in female and male *Cyp2c70* KO mice. This robust increase of these liver injury markers (Figs. 2 and S3) was successfully blocked with IBAT inhibition.

**Fig. 3:**
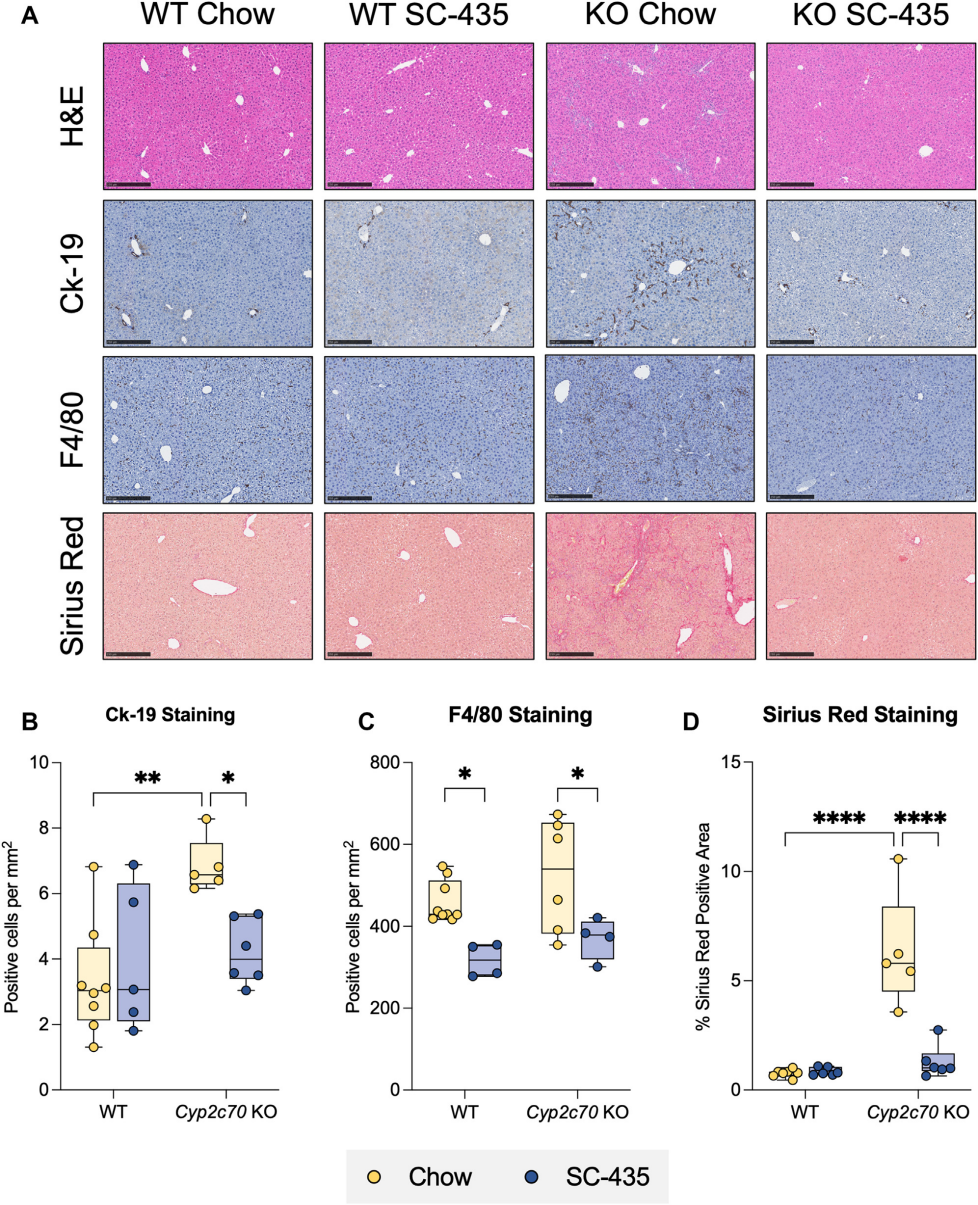
Immune and fibrotic responses in female *Cyp2c70* KO mice are alleviated with SC-435 treatment. A: Morphological response to SC-435 treatment in WT and *Cyp2c70 KO* mice. From top to bottom panel: Hematoxylin and eosin (H&E), Cytokeratin-19 (Ck-19), F4/80, and Sirius Red–stained liver sections (original magnification 10X) from the indicated genotypes and treatments groups. *Scale bar*, 250 μm. Quantification of positive B: Ck-19, C: F4/80 cells per mm^2^, and D: Percent Sirius Red positive area of the entire liver section. Asterisks indicate significant differences between groups. Median values (*line*), interquartile range (*boxes*), and min to max values are shown (*whiskers*) (∗*P* < 0.05, ∗∗*P* < 0.01, ∗∗∗∗*P* < 0.0001); n = 5–9 mice per group.

**Fig. 4:**
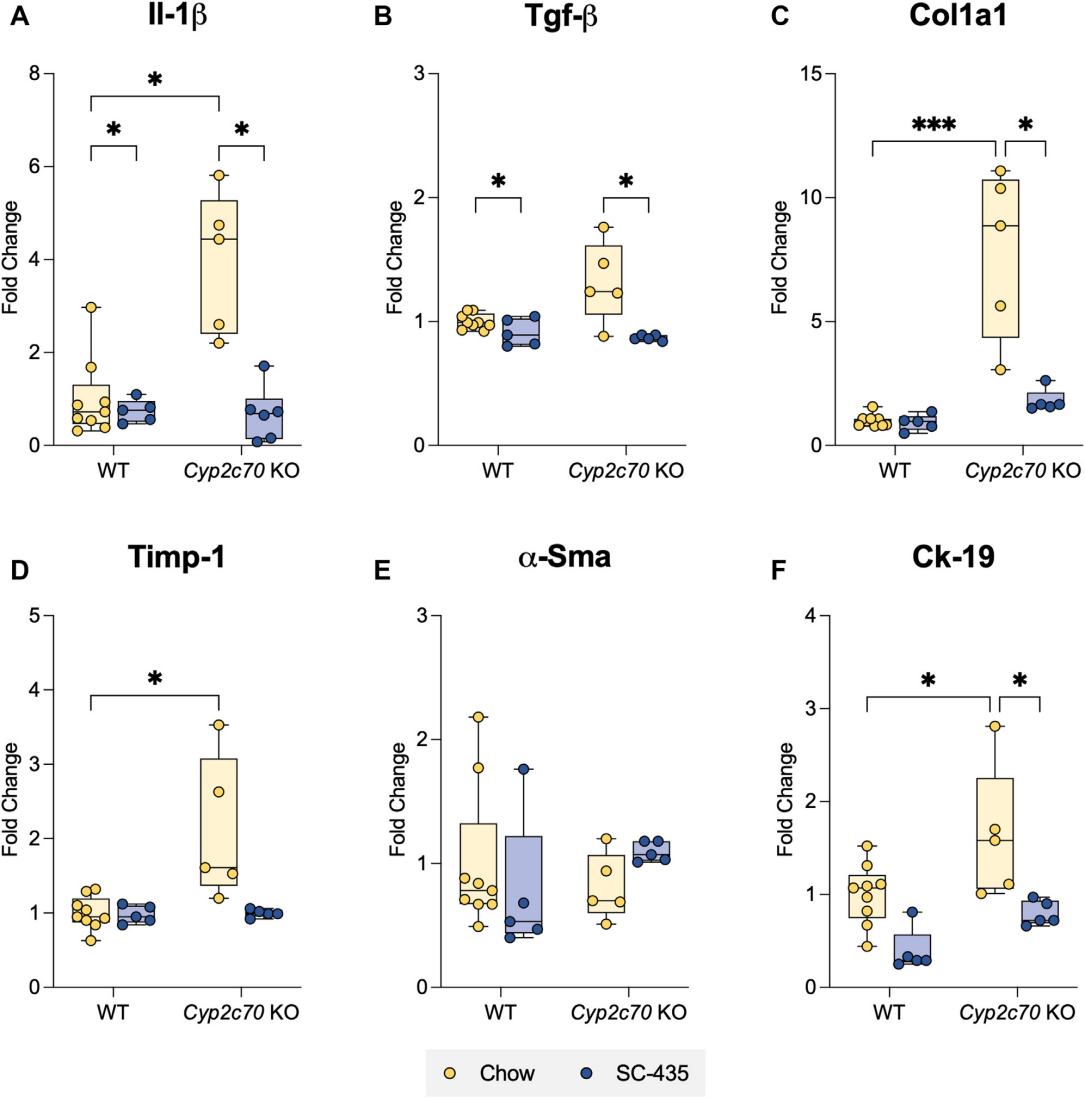
Increased hepatic expression of inflammation and fibrosis-related genes in female *Cyp2c70* KO mice is alleviated with SC-435 treatment. A: Il-1β, B: Tgf-β, C: Col1a1, D: Timp-1, E: α-Sma, and F: Ck-19 gene expression. RNA was isolated from the livers of individual mice and used for real-time PCR analysis. The mRNA expression was normalized using cyclophilin and the results for each gene are expressed relative to chow-fed WT mice. Asterisks indicate significant differences between groups. Median values (*line*), interquartile range (*boxes*), and min to max values are shown (*whiskers*) (∗*P* < 0.05, ∗∗∗*P* < 0.001); n = 5–9 mice per group. Il-1β, interleukin 1 beta; Tgf-β, transforming growth factor beta; Col1a1, type I collagen; Timp-1, tissue inhibitor matrix metalloproteinase-1; α-Sma, α smooth muscle alpha-actin.

At 12 weeks of age, female *Cyp2c70* KO mice exhibited many characteristic histological features of cholestatic liver injury, as measured by immunohistochemistry for Ck-19 delineating ductular reaction and F4/80 for macrophage infiltration and Sirius Red staining for collagen deposition (Fig. 3A). QuPath-mediated analysis of the liver images revealed that IBAT inhibition attenuated the increase in biliary cell proliferation (Ck-19, Fig. 3B), macrophage infiltration (F4/80, Fig. 3C), and fibrosis (Sirius Red, Fig. 3D) observed in *Cyp2c70* KO mice to levels indistinguishable from WT mice. Findings for the male cohort of *Cyp2c70* KO mice were similar; however, the magnitude of the histological changes was less pronounced than that observed for female *Cyp2c70* KO mice (supplemental Fig. S4A, D). To complement the histological findings, expression of marker genes for hepatic inflammation, fibrosis, and biliary proliferation were measured. Quantitative real time PCR measurements revealed that liver mRNA levels of interleukin 1 beta (Fig. 4A), transforming growth factor beta (Fig. 4B), type I collagen (Fig. 4C), tissue inhibitor matrix metalloproteinase-1 (Timp-1, Fig. 4D), and Ck-19 (Fig. 4F) were significantly elevated in female *Cyp2c70* KO mice and restored to WT levels with IBAT inhibition. In contrast, no significant change was observed in mRNA levels of α smooth muscle alpha-actin (Fig. 4E). In the male cohort of *Cyp2c70* KO mice, liver mRNA levels for transforming growth factor beta, tissue inhibitor matrix metalloproteinase-1, and Ck-19 were significantly increased, with a trend toward increased *Col1al* mRNA, and no significant changes observed in the mRNA levels of interleukin 1 beta and α smooth muscle alpha-actin. As in the female cohort, IBAT inhibition blocked the increase in expression of these liver injury markers in the male *Cyp2c70* KO mice (supplemental Fig. S5A, F).

### IBAT inhibition reduces expression of inflammatory pathway genes

**Fig. 5:**
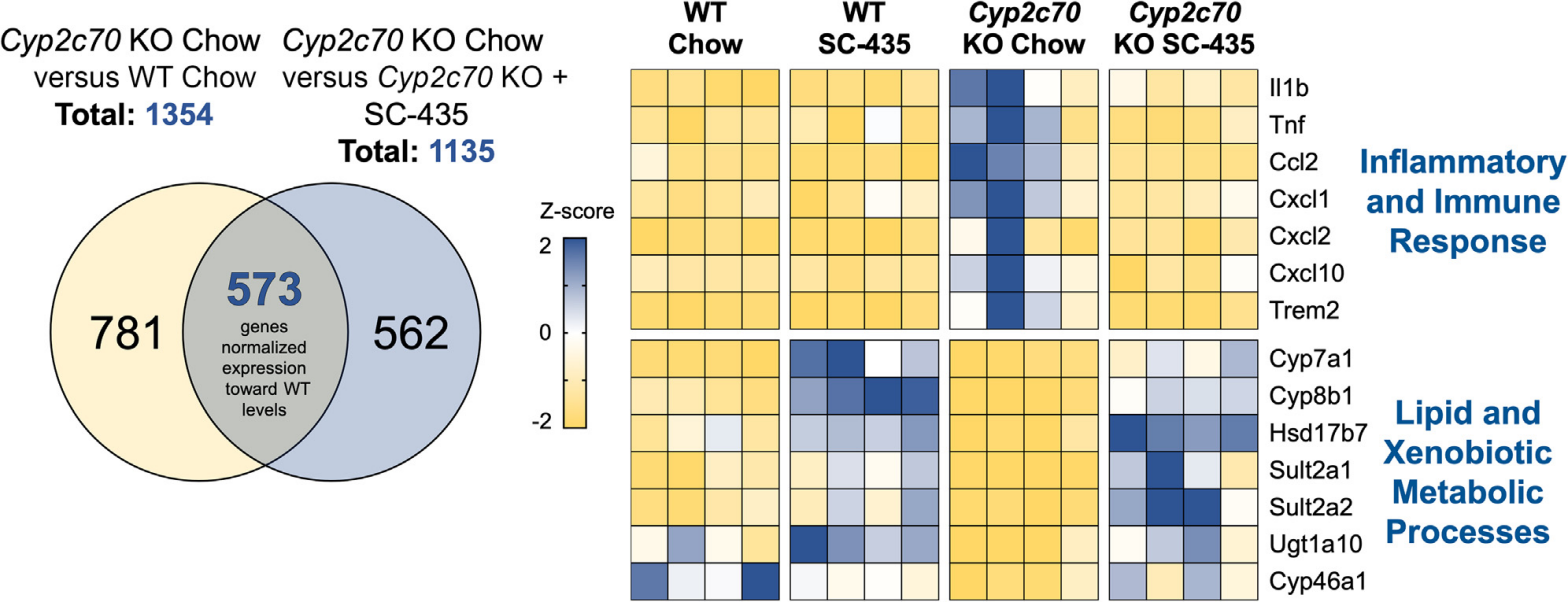
Female mouse liver RNA-Seq analysis. Venn diagram showing the number of differentially expressed genes identified in *Cyp2c70 KO* mice versus WT and *Cyp2c70* KO mice–fed chow versus chow plus SC-435. Intersection represents the subset of genes (488 downregulated plus 85 upregulated) whose expression is normalized toward WT levels. Change in expression is displayed as change in Z-score on heatmap for representative genes from the indicated pathways that were normalized with SC-435 treatment. Each column in the heatmap represents an individual animal.

To identify pathways that may underlie the IBATi’s therapeutic benefit, RNA-Seq analysis was performed using livers from female and male WT and *Cyp2c70* KO mice–fed chow or SC-435-containing chow diet (Fig. 5). Identification of differentially expressed genes with a log2 (fold-change) > 1 and adjusted *P* < 0.05 in female chow-fed *Cyp2c70* KO versus chow-fed WT mice revealed 216 genes were downregulated and 1138 genes were upregulated in *Cyp2c70* KO versus WT mice (total of 1354 differentially expressed genes). Using the same approach, female *Cyp2c70* KO mice–fed chow versus SC-435 were also compared. In that analysis, 721 genes were downregulated and 414 genes were upregulated in *Cyp2c70* KO mice by the SC-435 treatment (1135 total differentially expressed genes).

Gene set enrichment analysis was used to identify potential pathways that mediate hepatoprotective actions of the IBATi. We used KEGG analysis and the Database for Annotation, Visualization, and Integrated Discovery (DAVID) to annotate the genes to the pathway level. The absence of *Cyp2c70* significantly upregulated 48 pathways and downregulated 13 pathways when compared to WT by KEGG analysis (supplemental Fig. S6). IBAT inhibition in *Cyp2c70* KO mice downregulated 37 pathways and upregulated 6 pathways when compared to chow-fed *Cyp2c70* KO mice. To further understand the gene expression changes associated with reversal of *Cyp2c70* KO-associated liver injury, the genes upregulated in *Cyp2c70* KO versus WT mice (1138 genes) and downregulated in *Cyp2c70* KO mice–fed SC-435 versus chow (721 genes) were identified and compared. This analysis identified 488 genes whose expression decreased toward WT levels with SC-435 treatment and were largely related to pathways associated with inflammatory and immune response (Fig. 5). Expression of genes for inflammatory cytokines commonly found elevated in cholestasis such as *Ccl2*, *Cxcl1*, *Cxcl2*, and *Cxcl10* (35) was ameliorated by SC-435 treatment. The same approach was taken to narrow the list of genes downregulated in *Cyp2c70* KO versus WT mice (216 genes) and those upregulated in *Cyp2c70* KO mice–fed SC-435 versus chow (414 genes). This identified 85 genes whose expression increased toward WT levels with SC-435 treatment and were largely related to lipid and xenobiotic metabolic processes. In addition to BA biosynthetic genes, cholesterol 7α-hydroxylase (*Cyp7a1*) and sterol 12α-hydroxylase (*Cyp8b1*), genes encoding enzymes that contribute to sulfation and metabolism of steroids and BAs in the liver such as *Hsd17b7*, *Sult2a1,* and *Sult2a2* were significantly elevated in IBATi-treated animals. Because of the observed sex differences in the hepatic injury phenotype in this model, analysis of the liver gene expression changes in male mice was performed separately (supplemental Fig. S7). In the male mice, examination of genes upregulated in *Cyp2c70* KO versus WT mice (778 genes) and those downregulated in *Cyp2c70* KO mice in response to SC-435 feeding (1188 genes) revealed 563 common genes, which were largely related to pathways associated with inflammatory and immune response. Examination of genes downregulated in *Cyp2c70* KO versus WT mice (172 genes) and those upregulated in *Cyp2c70* KO mice in response to SC-435 feeding (391 genes) revealed 90 common genes, which were largely related to pathways related to lipid and xenobiotic metabolic processes. Common pathways pointing toward alleviating inflammation were found and changes in hepatic gene expression in the male group are shown in supplemental Fig. S8.

### IBAT inhibition alters BA metabolism and reduces hepatic BA content in *Cyp2c70* KO mice

**Fig. 6:**
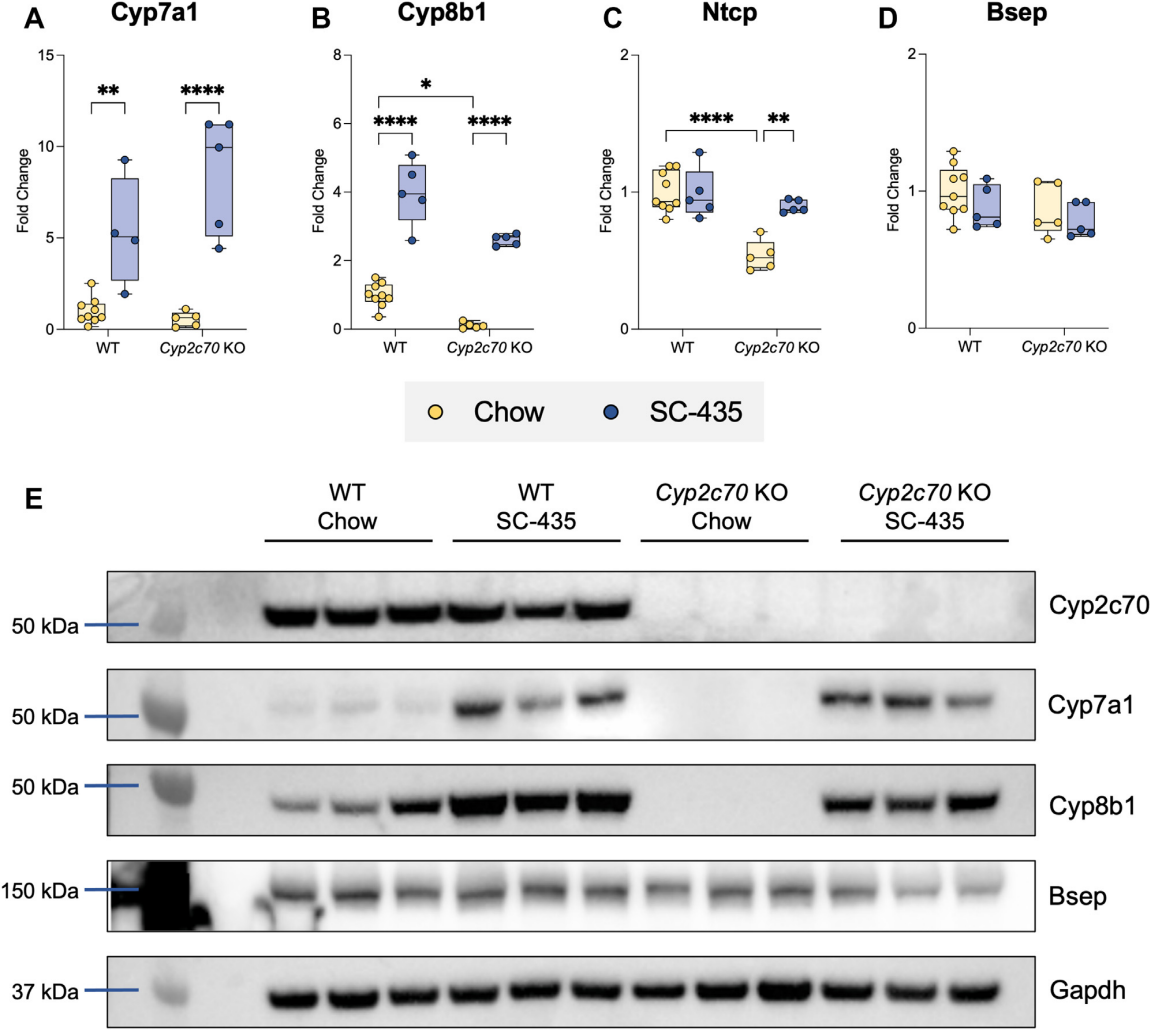
IBAT inhibition increases hepatic expression of BA metabolism genes in female WT and *Cyp2c70* KO mice. RNA was isolated from livers of individual mice and used for real-time PCR analysis. The mRNA expression was normalized using cyclophilin and the results for each gene are expressed relative to chow-fed WT mice. A: Cyp7a1; B: Cyp8b1; C: Ntcp; D: Bsep. Asterisks indicate significant differences between groups. Median values (*line*), interquartile range (*boxes*), and min to max values are shown (*whiskers*) (∗*P* < 0.05, ∗∗*P* < 0.01, ∗∗∗∗*P* < 0.0001); n = 5–9 mice per group. E: Protein was isolated from livers of individual mice and used for Western Blot analysis, n = 3 mice per group. BA, bile acid; IBAT, ileal BA transporter; Bsep, bile salt export pump; Ntcp, Na+-taurocholate co-transporting polypeptide.

To investigate the mechanisms underlying the hepatoprotective effects of IBAT inhibition in *Cyp2c70* KO mice, quantitative real-time PCR and immunoblotting were used to measure mRNA and protein expression of selected liver genes important for BA homeostasis and liver BA composition. Hepatic mRNA and protein expression of *Cyp7a1* and particularly *Cyp8b1* are decreased in female *Cyp2c70* KO versus WT mice (Fig. 6A, B). Treatment with the IBATi significantly increased hepatic *Cyp7a1* and *Cyp8b1* expression in all groups. This was particularly pronounced in *Cyp2c70* KO mice, where *Cyp8b1* mRNA and protein expression increased more than 10-fold with IBAT inhibition (Fig. 6E). In hepatocytes, BAs are taken up across the sinusoidal membrane by sodium-dependent and sodium-independent mechanisms involving the Na^+^-taurocholate cotransporting polypeptide (*Slc10a1*) and members of the Organic Anion Transport Protein (Oatp) family, respectively, whereas the bile salt export pump (*Abcb11*) mediates hepatocyte BA secretion across the canalicular membrane. Expression of Na^+^-taurocholate cotransporting polypeptide mRNA was reduced in *Cyp2c70* KO mice and restored to WT levels with IBAT inhibition (Fig. 6C). In contrast, bile salt export pump mRNA and protein expression were not significantly altered in *Cyp2c70* KO mice or in response to IBATi treatment (Fig. 6D, E). Qualitatively similar changes were observed in the male cohort of *Cyp2c70* KO mice, although the decrease in hepatic *Cyp8b1* expression is significantly attenuated in male versus female *Cyp2c70* KO mice. However, there were no sex-specific differences in the response to IBATi treatment at the mRNA (Figs. 6A–D and S9A–D) or protein expression (Figs. 6E and S9E) levels for these genes.

**Fig. 7:**
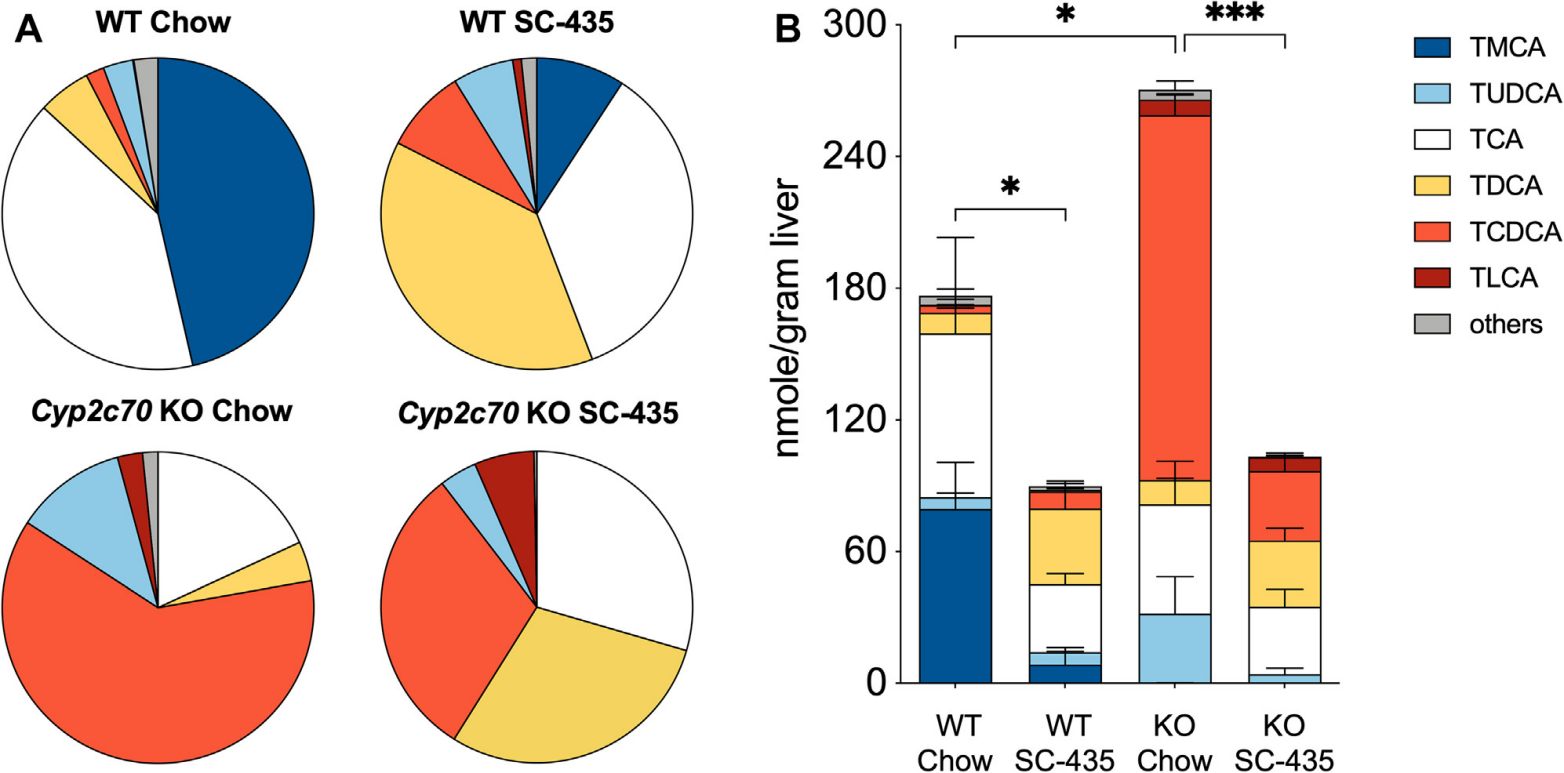
IBAT inhibition prevents the increase in liver BA retention in female *Cyp7c70* KO mice. A: Muricholates are absent in *Cyp2c70* KO mice. Pie charts for the liver BA profiles. B: Absence of *Cyp2c70* increased the total amount of liver-associated BAs and the amount of taurochenodeoxycholic acid (TCDCA) in *Cyp2c70* KO mice. Mean values ± SD are shown (∗*P* < 0.05, ∗∗∗*P* < 0.001); n = 4 mice per group. BA, bile acid; IBAT, ileal BA transporter.

**Fig. 8:**
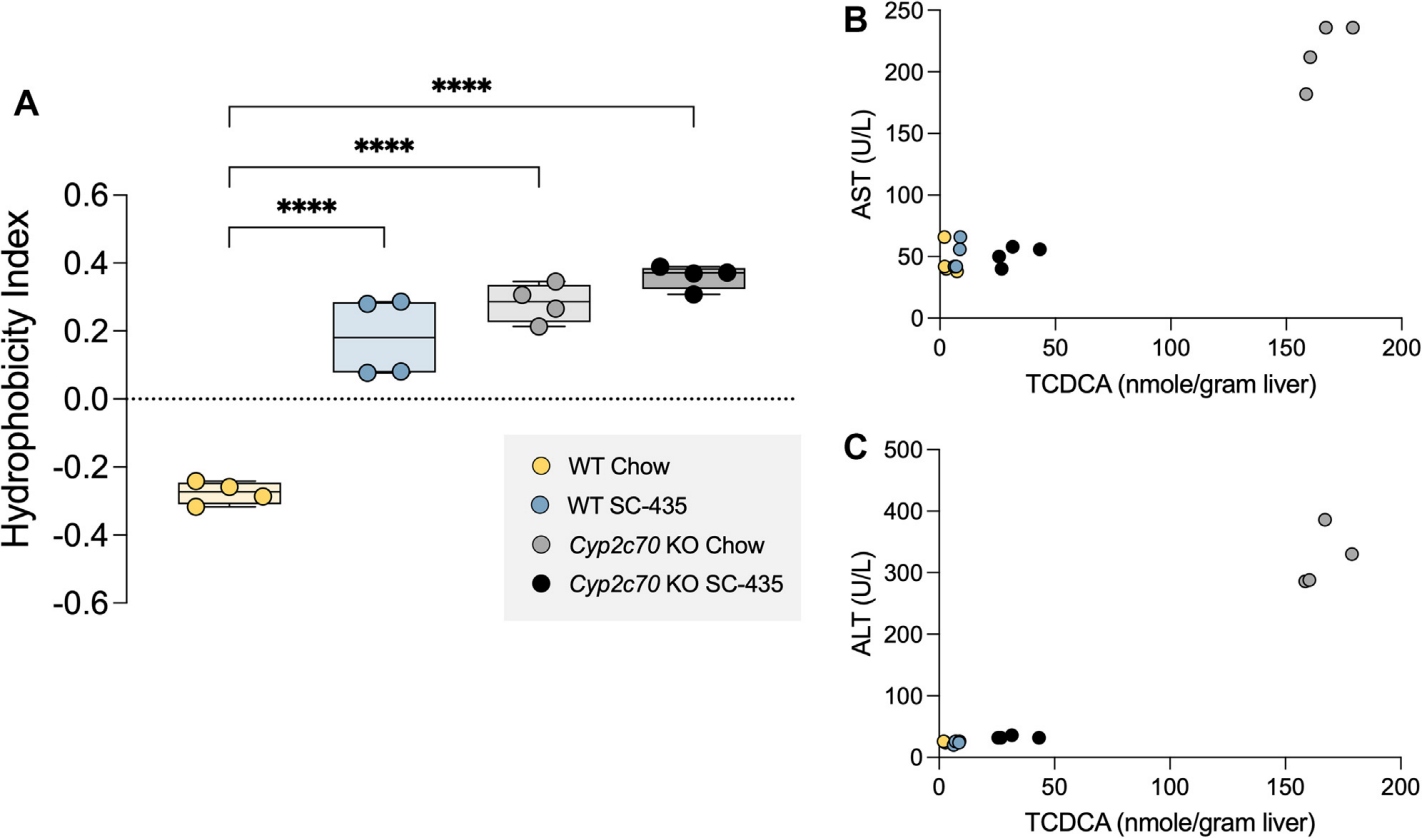
IBAT inhibition does not reduce liver BA hydrophobicity in female *Cyp7c70* KO mice. A: Liver BA composition was used to calculate the hydrophobicity index. IBAT inhibition and inactivation of *Cyp2c70* increases the hydrophobicity of the liver BA pool. Asterisks indicate significant differences between groups. Median values (*line*), interquartile range (*boxes*), and min to max values are shown (*whiskers*) (∗∗∗∗*P* < 0.0001); n = 4 mice per group. B and C: Liver amount of taurochenodeoxycholic acid (TCDCA) and serum levels of alanine aminotransferase (ALT) and aspartate aminotransferase (AST) are reduced in IBAT inhibitor–treated *Cyp2c70* KO mice. BA, bile acid; IBAT, ileal BA transporter.

To determine how IBAT inhibition affected liver BA composition and content in the *Cyp2c70* KO mice, liver extracts were prepared and analyzed by HPLC-electrospray ionization mass spectrometry (36). The pool of liver-associated BAs included primarily taurine conjugates, with unconjugated BAs constituting less than ∼2% of the total in all groups. The relative proportion of individual BA species and their contribution to the liver total BA content for female mice are shown in Fig. 7A, B, respectively. The concentrations (nmol/g liver) for the individual BA species measured are shown in supplemental Table S3. In addition to being devoid of MCAs, *Cyp2c70* KO mouse livers are enriched in taurochenodeoxycholic acid (TCDCA) and have an increased amount of tauroursodeoxycholic acid (TUDCA). This increase in TUDCA is likely due to several mechanism including the following: 1) the increased production of CDCA, which can be converted to 7-keto-lithocholic acid by gut bacterial 7αhydroxysteroid dehydrogenases and then reduced to UDCA by gut bacterial 7βhydroxysteroid dehydrogenases or the hepatocyte enzyme 11β-hydroxysteroid dehydrogenase1 (37, 38) and 2) the loss of Cyp2c70-mediated conversion of UDCA to βMCA (13, 14, 17, 39). The levels of taurolithocholic acid were also significantly increased in female *Cyp2c70* KO mouse livers but constituted less than 3% of the total BAs. The overall effect is to significantly increase the hydrophobicity of the liver BAs, with the calculated hydrophobicity index (HI) (40) value increasing from -0.28 in WT mice to +0.28 in *Cyp2c70* KO mice (Fig. 8A). In agreement with previous reports (24), IBAT inhibition reduced the proportion of hydrophilic tauromuricholic acids and increased taurodeoxycholic acid (TDCA) and TCDCA in livers of WT mice, thereby elevating the calculated HI from -0.28 to +0.18. In *Cyp2c70* KO mice, IBAT inhibition reduced the proportion of TCDCA plus TUDCA, increased the proportion of taurocholic acid (TCA) plus TDCA, and further increased the calculated HI of the liver-associated BAs to +0.36. To reconcile how IBAT inhibition can both increase hydrophobicity of the hepatic BA content and reduce liver injury in *Cyp2c70* KO mice, the total liver BA content was compared between groups. Hepatic BA content in chow-fed female *Cyp2c70* KO was significantly increased by 53% versus WT mice (Fig. 7B). Despite increases in hepatic Cyp7a1 expression and a predicted increase in hepatic BA synthesis, treatment with the IBATi to interrupt the BA enterohepatic circulation significantly reduced the total liver BA content by 49% and 62% in female WT and *Cyp2c70* KO mice, respectively. The IBATi-associated improvement in liver histology and serum markers of injury and decrease in liver BA content in *Cyp2c70* KO mice in the absence of a reduction in the overall hydrophobicity of the liver BAs suggests that reducing the hepatic BA accumulation may be sufficient to alleviate the injury. The hepatic toxicity of CDCA has been well documented in animal models, and levels of liver-associated TCDCA were reduced along with the serum transaminases in the IBATi-treated *Cyp2c70* KO mice (Figs. 8B, C). The liver BAs in male mice showed the same trends with inactivation of *Cyp2c70* and the response to IBAT inhibition as the female mice (supplemental Fig. S10 and supplemental Table S4). The hepatic BA content in chow-fed male *Cyp2c70* KO was increased by 65% versus WT mice and reduced with IBATi treatment by 56% and 54% in the WT and *Cyp2c70* KO mice, respectively. Total liver BA levels were lower in male versus female *Cyp2c70* KO mice, in agreement with the milder liver injury phenotype. However, hydrophobicity of the liver BAs was similarly elevated in male and female *Cyp2c70* KO versus WT mice and in male and female IBATi-treated versus untreated mice (supplemental Fig. S11). The calculated hydrophobicity values were -0.31 and +0.23 in chow and IBATi-treated WT male mice, respectively, and +0.25 and +0.34 in chow and IBATi-treated male *Cyp2c70* KO mice. As in the female mice, the amount of liver-associated TCDCA and the serum levels of ALT and AST were reduced in the IBATi-treated male *Cyp2c70* KO mice (supplemental Fig. S11).

### Detergency of the hepatic BA pool in *Cyp2c70* KO mice

**Fig. 9:**
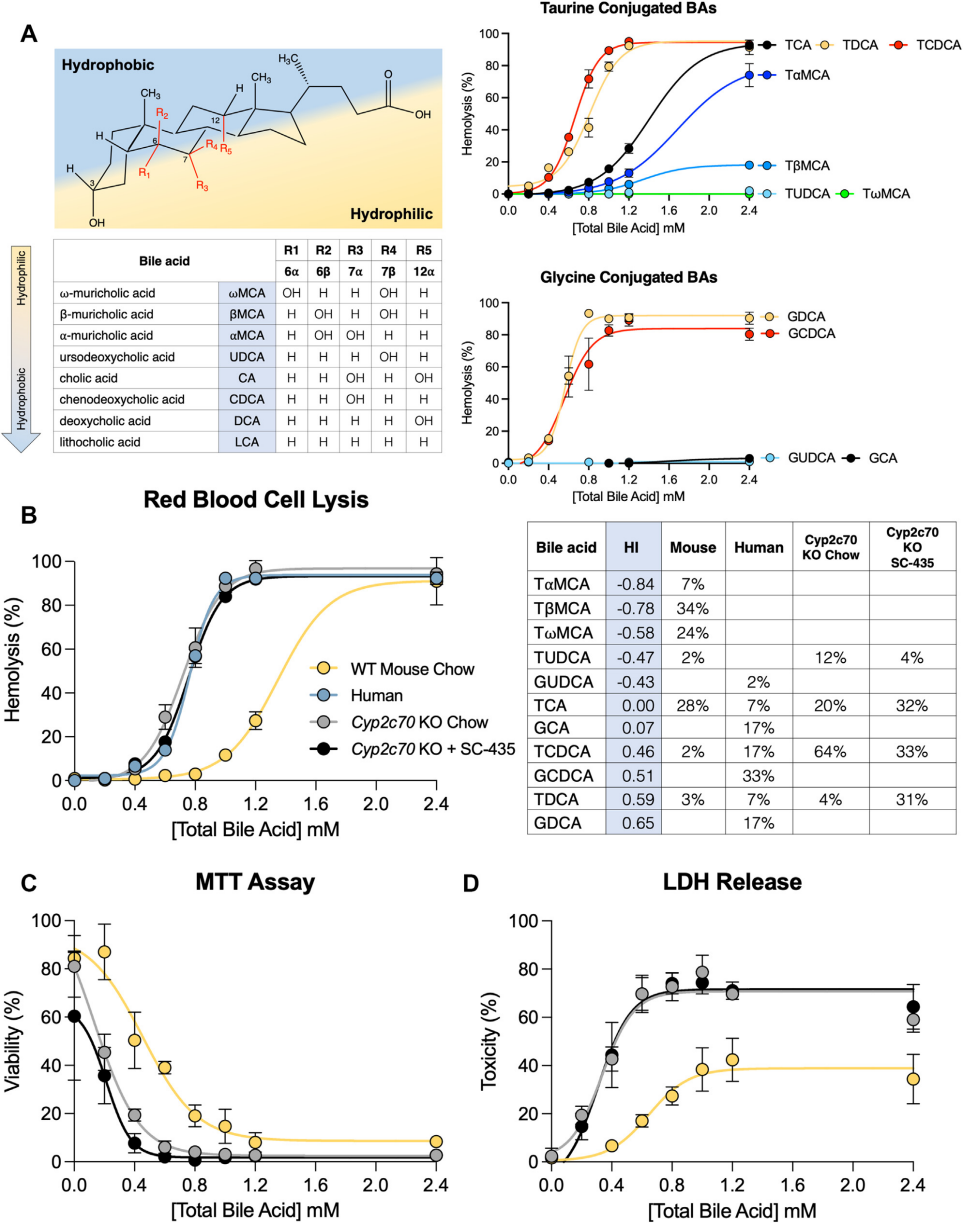
The liver BAs of *Cyp2c70* KO mice are more cytotoxic and resemble a human-like composition. A: The structure, common name, position of the hydroxyl groups, relative hydrophobicity, and hemolysis potential of the individual major BAs commonly found in mammals. Red blood cells isolated from mouse whole blood were incubated with the indicated concentrations of BAs for 30 min at 37°C and the amount of hemolysis was measured. B: Composition and hemolysis profile of liver BAs in female WT and *Cyp2c70* KO mice–fed chow or chow plus SC-435 as compared to human. The calculated hydrophobicity index (HI) and compositions of the BA mixtures used for the incubations are shown. C: AML12 (alpha mouse liver 12) cells were incubated with the indicated concentration of BA mixtures modeling the liver BA composition of WT or *Cyp2c70* KO mice and cell viability was quantified by MTT assay. D: AML12 cells were incubated with the indicated concentration of BA mixtures and BA toxicity measured by lactate dehydrogenase (LDH) release. Mean values ± SD are shown. BA, bile acid.

To complement the in vivo findings, the ability of individual BAs and BA mixtures to induce membrane damage and toxicity was assessed. The in vitro toxicity of BAs generally correlate with their detergency, which is concentration dependent and reflects their ability to self-aggregate (41). As a measurement of detergency and the potential for membrane lipid bilayer damage, the ability of individual conjugated BAs to lyse RBCs was evaluated. Whereas this model system has been used previously to study individual BAs from a variety of species (42), cytolytic properties of the individual taurine-conjugated muricholate species, TαMCA, TβMCA, and TωMCA have not been reported. The detergency of individual taurine-conjugated BAs partially followed their hydrophobicity estimated from their reported relative retention time by HPLC using a C18 octadecylsilane stationary phase (40, 43) (Fig. 9A), with TCDCA ≥ TDCA >> TCA > TαMCA>> TβMCA >> TωMCA = TUDCA. Despite having a 1-octanol-water partition coefficient that is identical to TCDCA (44), TUDCA shows no hemolytic activity over the concentration range examined. Interestingly, TαMCA and TβMCA showed cytolytic activity that was intermediate between TUDCA and TCA, whereas TωMCA, microbial-derived 6α-epimer of TβMCA, exhibited an RBC lytic activity similar to TUDCA and failed to induce hemolysis in this assay. For comparison, the RBC lytic activity of a limited number of individual glycine-conjugated BAs are shown (Fig. 9A) with GDCA > GCDCA >> GCA = GUDCA. To examine the detergency and toxicity properties of the BA compositions in the WT and *Cyp2c70* KO mice–fed chow and IBATi, the RBC assay was performed using mixtures of BAs approximating the measured hepatic BA compositions shown in Fig. 7. As expected, the WT mouse liver BA composition, which contains almost 50% hydrophilic muricholates, exhibits weak cytolytic activity. Mixtures modeling the hepatic BA composition of female *Cyp2c70* KO mice–fed chow or chow-containing IBATi were approximately twice as potent in the RBC lysis assay and exhibit activity similar to a model human liver BA composition (45) (Fig. 9B). To complement these findings, two additional measurements of cellular toxicity were assessed. Similar results were obtained using the BA mixtures approximating the hepatic BA compositions for WT and *Cyp2c70* KO mice when measuring cellular viability and metabolic activity using a MTT assay (Fig. 9C) and cellular cytotoxicity using lactate dehydrogenase release (Fig. 9D).

## Discussion

The major finding of this study is that reduction of hepatic BA accumulation with IBAT inhibition protects *Cyp2c70* KO mice from hepatic injury, independent of the hydrophobicity of the BA pool. Although BAs have previously been implicated as a factor in cholestatic liver disease (3, 5, 6, 7), the precise mechanisms by which BAs and toxic bile contribute to the pathogenesis of human cholestatic liver disease remains a fundamental unresolved question (8, 9, 10). Identification of *Cyp2c70* as the hepatic cytochrome P450 enzyme responsible for the synthesis of muricholates by the Myomorpha suborder of rodents (mice, rats, voles, jerboas) (2) was a significant breakthrough in the field (13) and has enabled the generation and study of *Cyp2c70* KO mice as a laboratory model with a more human-like hydrophobic BA composition (14, 15, 17, 18). As previously reported by others, the *Cyp2c70* KO mice in this study lack hydrophilic 6-hydroxylated MCAs and possess a hepatic BA composition dominated by CDCA. This change in primary BA synthesis by *Cyp2c70* KO mice was associated with increases in liver and spleen weight, elevation of serum markers of liver damage, and histological evidence of liver injury including ductular reaction, immune cell infiltration, and fibrosis. The liver injury phenotype was more pronounced in female versus male *Cyp2c70* KO mice. This observation has been previously reported and was attributed in part to a greater reduction in hepatic *Cyp8b1* expression and reduced synthesis of CA, a more hydrophilic BA, in the female versus male *Cyp2c70* KO mice (18).

In addition to a more hydrophobic CDCA-enriched BA composition, liver BA content has been reported to be elevated in *Cyp2c70* KO versus WT mice (14, 15). This is despite a reduction in hepatic BA synthesis and the whole-body BA pool size (14, 18). The mechanisms responsible for the increased hepatic BA retention in *Cyp2c70* KO mice have not been identified but may include the lower bile secretory capacity for hydrophobic versus hydrophilic BAs (46, 47) or hydrophobic BA-induced damage to cytoplasmic organelles (3, 48), the canalicular membrane (49, 50, 51), intrahepatic ductules (Canals of Hering), or bile ducts (52, 53). Whereas the increased hydrophobicity of the BA pool in *Cyp2c70* KO mice likely has a significant role in initiating the hepatocellular damage, persistent elevated intrahepatic BA levels may play a role in driving the progression of hepatocellular injury. To further understand the mechanisms underlying the liver injury in *Cyp2c70* KO mice, we studied the effects of pharmacological inhibition of the ileal BA transporter, an intervention that has previously been shown to reduce liver BA concentrations and yield benefit in the *Mdr2* KO mouse model of cholestatic and bile duct injury (8, 19).

IBAT inhibition significantly increases the hepatic expression of *Cyp7a1* and *Cyp8b1* in WT and *Cyp2c70* KO mice. This was particularly evident for *Cyp8b1* in female *Cyp2c70* KO mice, resulting in a predicted shift in hepatic BA synthesis toward CA and away from CDCA. However, IBAT inhibition also significantly increases BA flux into the colon, where the BAs undergo bacterial deconjugation and 7α-dehydroxylation, thereby increasing the conversion of CA to DCA (54). Unconjugated dihydroxy BAs such as CDCA and DCA are sufficiently hydrophobic to undergo passive absorption in the colon (55, 56) and are transported back to the liver in the portal circulation for uptake, reconjugation, and resecretion into bile. Interestingly, *Cyp2a12* has recently been identified as the mouse liver enzyme responsible for the 7α-rehydroxylation of TDCA to TCA (14). However, as observed for previous studies of IBAT inhibition in mice (24), the increased return of DCA to the liver appears to overwhelm the ability of *Cyp2a12* to efficiently convert TDCA to TCA. As a result, there is an increase in the hepatic proportion of TDCA, a hydrophobic dihydroxy BA with detergency properties similar to TCDCA (57). In agreement with previous findings (14, 18), examination of the liver BA composition of *Cyp2c70* KO mice in this study revealed a significant increase in hydrophobic BA species and calculated hydrophobicity index. Hydrophobic BAs such as CDCA are known to induce liver toxicity, whereas hydrophilic bile acids like UDCA are rarely associated with liver damage (9, 58). In support of the hypothesis that the altered BA composition plays an important role in the liver injury phenotype, administration of UDCA to *Cyp2c70* KO mice has been shown to increase the hydrophilicity of biliary BAs, improve liver histology, reduce plasma BA and transaminases levels, and normalize the hepatic and intestinal function toward that of WT mice (18). This was also evident in *Cyp2c70*-*Cyp2a12* double KO mice, where UDCA feeding improved neonatal viability and was protective in the male and female double KO mice at 6 weeks of age and in male double KO mice at 20 weeks of age. (59). In contrast, UDCA-treated female *Cyp2c70*-*Cyp2a12* double KO mice at 20 weeks of age still exhibited significant liver injury, with a greatly elevated hepatic BA content and taurolithocholic acid–enriched hydrophobic BA composition (59). When the steroidal farnesoid X-receptor agonist obeticholic acid was recently evaluated in *Cyp2c70* KO mice, short-term 4-weeks treatment did not improve the hepatic fibrosis or development of cholangiopathy, despite suppressing BA synthesis (60). The lack of a beneficial effect was attributed to an increase in the hydrophobicity of biliary BAs in the obeticholic acid–treated *Cyp2c70* KO mice. Paradoxically, administration of an IBAT inhibitor to *Cyp2c70* KO mice in this study prevented the development of cholangiopathy and liver fibrosis, despite increasing the hydrophobicity of the liver BAs to HI values similar to those of serum and bile BA samples analyzed in the obeticholic acid–treated *Cyp2c70* KO mice (60). This is likely due to the significant reduction in the retention of liver-associated BAs, which are decreased more than 50% in the IBAT inhibitor–treated *Cyp2c70* KO mice. In our study, the markers of liver injury AST and ALT were elevated in *Cyp2c70* KO mice when hepatic levels of the hydrophobic BA TCDCA exceeded ∼50 nmol/g of liver. As a result of an efficient BA enterohepatic circulation, previously synthesized BAs that have been secreted into bile and reabsorbed from the intestine are thought to account for approximately 95% of the liver BA flux, with newly synthesized BAs constituting the remaining ∼5% (54, 61). Although hepatic BA synthesis is induced by interruption of the enterohepatic circulation, new synthesis is insufficient to account for a block in ileal active reabsorption (62). This may help explain the differences between our study and the reported findings for obeticholic acid–treated *Cyp2c70* KO mice (60). Hepatic BA content was not measured in that study, however, we hypothesize that obeticholic acid suppression of hepatic BA synthesis was not adequate to yield similar decreases in the hepatic BA content. Interestingly, IBAT inhibition has been shown to reduce hepatic BA retention and improve biomarkers of hepatocellular and cholestatic injury in *Mdr2* KO mice (8, 19), whereas a 4 or 6-weeks treatment of this model with obeticholic acid showed only limited therapeutic benefit (63, 64).

The development of cholangiopathy and liver fibrosis in *Cyp2c70* KO mice has been attributed to the lack of the 6-hydroxylated MCAs, which normally comprise upward of 40%–50% of the murine BA pool. Whereas the physicochemical properties of CA, DCA, CDCA, LCA, and their taurine and glycine conjugates have been the subject of considerable study (41, 44, 57) and the bactericidal activity of individual unconjugated MCAs have been reported (65), the detergency properties of taurine-conjugated MCAs or mixtures of conjugated MCAs and nontrihydroxylated BAs have not been described. Using the RBC lysis model as a measure of detergency, TαMCA and TβMCA demonstrated membrane lysis properties intermediate between TUDCA and the more hydrophobic BA species TCA, TDCA, and TCDCA. Whereas bacteria-mediated reactions such as 7α-dehydroxylation that convert primary to secondary BAs typically increases detergency, microbial epimerization of the 6-hydroxy group of βMCA to yield the murine major secondary BA ωMCA dramatically reduces its detergent properties. This may be due to the alpha-orientation of the 6-hydroxy group in ωMCA, which like the 7β-hydroxy group in UDCA is equatorial to the plane of the steroid nucleus and favors interaction with water (43, 66). Mixtures of BAs approximating the measured hepatic BA composition in *Cyp2c70* KO mice were remarkably similar to that reported for human liver in the RBC lysis assay, supporting the concept that while still imperfect, the *Cyp2c70* KO mice may more closely model BA effects in the pathogenesis of liver disease in humans.

In summary, IBAT inhibition improved cholangiopathy and the hepatic injury in *Cyp2c70* KO mice despite increasing hepatic BA synthesis and hydrophobicity of the hepatic BA pool. The improvement was associated with a reduction in the total hepatic BA accumulation. The findings suggest that in addition to the important role of hydrophobicity, there may be a therapeutically addressable threshold for hepatic BA-induced injury by targeting the return of BAs to the liver by inhibiting the function of the intestinal bile acid transporter. Further studies will be required to determine if similar mechanisms are responsible in part for the clinical benefit of IBAT inhibitors that was recently demonstrated in children with progressive familial intrahepatic cholestasis and Alagille syndrome (21, 22).

## Data availability

The liver RNA-Seq dataset is available from the GEO repository with the following accession number: GSE145020.

## Supplemental data

This article contains supplemental data.

## Conflict of interest

One or more of the authors has an actual or perceived conflict of interest with the contents of this article. Paul A. Dawson received Research Funding from Albireo Pharma. Saul J. Karpen received Consulting fees from Albireo Pharma, Intercept Pharmaceutical, and Mirum Pharma.
