# Supplementary material for: Ileal bile acid transporter inhibition in Cyp2c70 KO mice ameliorates cholestatic liver injury
Source: J Lipid Res. 2022 Aug 5;63(9):100261. doi: 10.1016/j.jlr.2022.100261 (PMC9460185; doi:10.1016/j.jlr.2022.100261)
Supplement: Supplemental Information [file mmc1.pdf]

Supplemental Data

Ileal bile acid transporter inhibition in *Cyp2c70* KO mice ameliorates cholestatic liver injury

Jennifer K. Truong[1], Ashley L. Bennett[1], Caroline Klindt[1], Ajay C. Donepudi[1], Sudarshan R. Malla[1], Kimberly J. Pachura[1], Alex Zaufel[2], Tarek Moustafa[2], Paul A. Dawson[1], and Saul J. Karpen[1]

[1]Department of Pediatrics, Division of Pediatric Gastroenterology, Hepatology and Nutrition, Emory University School of Medicine, Children's Healthcare of Atlanta, Atlanta, Georgia, United States [2]Division of Gastroenterology and Hepatology, Department of Internal Medicine, Medical University of Graz, Graz, Austria

Supplemental Methods

Supplemental Table S1: Mouse primer sets used for RT-PCR analysis

**Gene name**	**Forward primer 5'-3'**	**Reverse primer 5'-3'**
Col1a1	TAGGCCATTGTGTATGCAGC	ACATGTTCAGCTTTGTGGACC
Tgf-β	CACCGGAGAGCCCTGGATA	TGTACAGCTGCCGCACACA
IL-1β	CAACCAACAAGTG ATATTCTCCATG	GATCCACACTCTC CAGCTGCA
Timp1	AGGTGGTCTCGTTGATTTCT	GTAAGGCCTGTAGCTGTGCC
α-SMA	GTTCAGTGGTGCCTCTGTCA	ACTGGGACGACATGGAAAAG
Cyp8b1	TTCGACTTCAAGCTGGTCGA	CAAAGCCCCAGCGCCT
Bsep	CTGCCAAGGATGCTAATGCA	CGATGGCTACCCTTTGCTTC
Ntcp	ATGACCACCTGCTCCAGCTT	GCCTTTGTAGGGCACCTTGT
Cyp7a1	CAGGGAGATGCTCTGTGTTCA	AGGCATACATGCAAAACCTCC
Ck-19	CCGGACCCTCCCGAGATTA	CTCCACGCTCAGACGCAAG
Cyclophilin	GGCCGATGACGAGCCC	TGTCTTTGGAACTTTGTCTGCA

Supplemental Table S2: Source of the primary and secondary antibodies used in the study

Antibody	Catalog #	Dilutions	Vendor
Bsep	PA5-78690	1:1000	Invitrogen
Cyp7a1	PA5-100892	1:1000	Invitrogen
Cyp8b1	PA5-37088	1:1000	Invitrogen
Cyp2c22/70		1:3000	Research Gift from Drs. Eddy Morgan, Choon-Myung Lee (Emory University)
Gapdh	MA5-15738	1:5000	Invitrogen
Goat anti-mouse IgG-HRP	sc-2005	1:5000	Santa Cruz
Goat anti-rabbit IgG-HRP	sc-2064	1:5000	Santa Cruz
Ck-19	ab52625	1:500	Abcam
F4/80	70076	1:100	Cell Signaling

Supplemental Table Legends

	**WT Chow**	**WT SC-435**	***Cyp2c70* KO Chow**	***Cyp2c70* KO SC-435**
TαMCA	3.76 ± 3.11[a]	2.13 ± 1[a]	BLD	BLD
TβMCA	26.52 ± 11.49[a]	2.51 ± 1.62[a]	BLD	BLD
TωMCA	49.13 ± 9[a]	3.91 ± 3.55[b]	BLD	BLD
TCA	74.71 ± 43.73[a]	30.96 ± 4.68[a]	49.73 ± 19.68[a]	30.75 ± 7.84[a]
TDCA	9.33 ± 2.09[a]	34.49 ± 12.51[b]	11.15 ± 0.77[a]	30.11 ± 5.63[b]
TCDCA	3.54 ± 2.62[a]	7.72 ± 1.22[a]	166.27 ± 9.13[b]	31.79 ± 8.1[c]
TUDCA	5.37 ± 1.94[a]	5.68 ± 2.13[a]	31.71 ± 16.79[b]	4.21 ± 2.69[a]
TLCA	0.18 ± 0.07[a]	0.82 ± 0.48[a]	7.05 ± 2.68[b]	6.15 ± 0.98[b]
GCA	0.11 ± 0.05[a]	BLD	0.13 ± 0.04[a]	BLD
GCDCA	BLD	BLD	0.25 ± 0.03[a]	BLD
αMCA	0.18 ± 0.13[a]	0.15 ± 0.14[a]	BLD	BLD
βMCA	0.87 ± 0.43[a]	0.21 ± 0.19[b]	BLD	BLD
ωMCA	0.84 ± 0.61[a]	0.43 ± 0.49[a]	0.08 ± 0.04[a]	BLD
CA	1.98 ± 2.01[a]	0.52 ± 0.41[a]	2.39 ± 2.77[a]	0.26 ± 0.12[a]
DCA	BLD	0.12 ± 0.03[a]	BLD	0.08 ± 0.03[a]
CDCA	BLD	BLD	0.96 ± 0.72[a]	0.08 ± 0.03[a]
UDCA	BLD	0.1 ± 0.07[a]	0.57 ± 0.5[a]	BLD

Table S3. Female mouse liver BA composition. BAs were extracted from liver homogenate and analyzed using electrospray ionization mass spectrometry. BA concentrations (nmol/g liver) are reported as mean ± SD; n = 4 mice per group. Values with different superscript letters are significantly different ($P < 0.05$) according to ordinary two-way ANOVA and Sidak's multiple comparisons test or T-test when appropriate. BLD = below level of detection.

	WT Chow	WT SC-435	*Cyp2c70* KO Chow	*Cyp2c70* KO SC-435
TαMCA	5.99 ± 5.95[a]	0.11 ± 0.13[a]	0.58 ± 0.16[a]	0.15 ± 0.19[a]
TβMCA	24.48 ± 24.95[a]	1.15 ± 0.52[a]	0.35 ± 0.18[a]	0.25 ± 0.15[a]
TωMCA	0.06 ± 0.05[a]	0.02 ± 0.03[a]	0.8 ± 0.62[b]	BLD
TCA	26.24 ± 21.48[a]	10.17 ± 4.36[a]	23.5 ± 2.98[a]	10.96 ± 5.09[a]
TDCA	4.37 ± 2.91[a]	13.3 ± 8.9[a]	0.18 ± 0.03[b]	6.14 ± 3.06[a,b]
TCDCA	0.42 ± 0.25[a]	1.09 ± 0.34[a]	68.37 ± 38.09[b]	26.54 ± 16.86[a,b]
TUDCA	1.47 ± 1.37[a]	1.71 ± 1.37[a]	9.29 ± 6.57[b]	1.23 ± 0.85[a]
TLCA	0.03 ± 0.02[a]	0.13 ± 0.15[a]	1.77 ± 1.11[a]	2.07 ± 1.93[a]
GCA	BLD	BLD	BLD	BLD
GCDCA	BLD	BLD	BLD	BLD
αMCA	BLD	BLD	BLD	BLD
βMCA	0.23 ± 0.45	BLD	BLD	BLD
ωMCA	BLD	BLD	BLD	BLD
CA	0.19 ± 0.38	BLD	BLD	BLD
DCA	BLD	BLD	BLD	BLD
CDCA	BLD	BLD	BLD	BLD
UDCA	BLD	BLD	BLD	BLD
LCA	BLD	0.18 ± 0.21[a]	BLD	0.42 ± 0.11[b]

Table S4. Male mouse liver BA composition. BAs were extracted from liver homogenate and analyzed using electrospray ionization mass spectrometry. BA concentrations (nmol/g liver) are reported as mean ± SD; n = 4 mice per group. Values with different superscript letters are significantly different ($P < 0.05$) according to ordinary two-way ANOVA and Sidak's multiple comparisons test or T-test when appropriate. BLD = below level of detection.

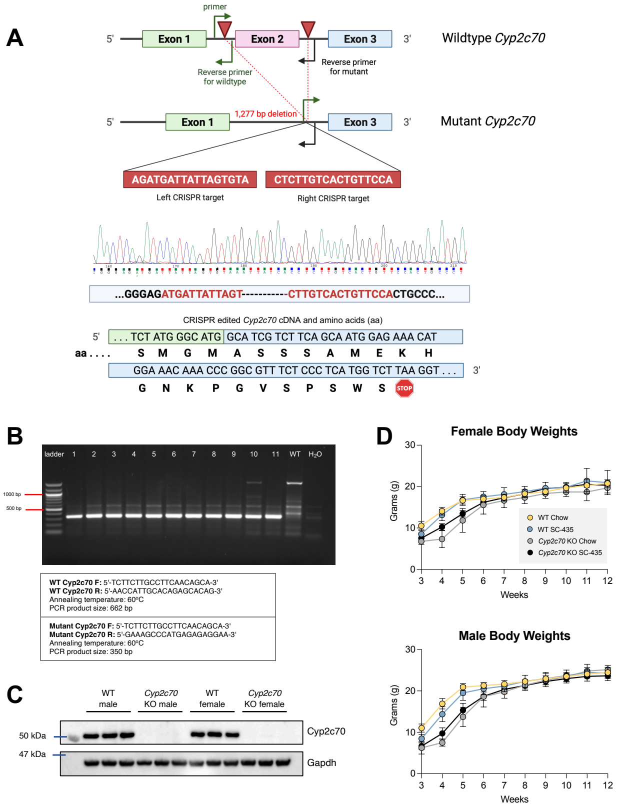

Fig. S1. Generation of *Cyp2c70* KO mouse. A: CRISPR target site in WT *Cyp2c70* and predicted cDNA and amino acid sequences of mutant *Cyp2c70*. Primers were designed for genotyping mice to detect the 1,277 bp deletion in *Cyp2c70* induced by Cas9. The indicated forward and reverse primers amplify the WT allele (662 bp) or mutant allele (350 bp). The predicted mutant *Cyp2c70* cDNA and protein, which arise from splicing of exon 1 to exon 3, are shown. The mutant allele encodes a predicted transcript with a frameshift at amino acid position 57 and premature stop at codon 78. The WT *Cyp2c70* encodes a 489 amino acid polypeptide whereas the predicted mutant *Cyp2c70* allele encodes a predicted 77 amino acid polypeptide with a unique 19 amino acid C-terminus. B: PCR genotyping of *Cyp2c70*$^{-/-}$, *Cyp2c70*$^{+/-}$, and WT mice (lanes 1 to 11). C: Immunoblotting of liver extracts from adult male and female WT and *Cyp2c70 KO* mice. Protein (0.25 µg) of liver detergent extract from individual mice were subjected to SDS-PAGE and immunoblotting using a rabbit anti-murine Cyp2c70 antibody. Predicted size of the Cyp2c70 protein is 56 kDa. D: Body weight of female and male WT and *Cyp2c70 KO* mice fed chow or chow plus SC-435 starting at 4 weeks of age. Body weight was monitored weekly. N = 5 – 9 mice per group.

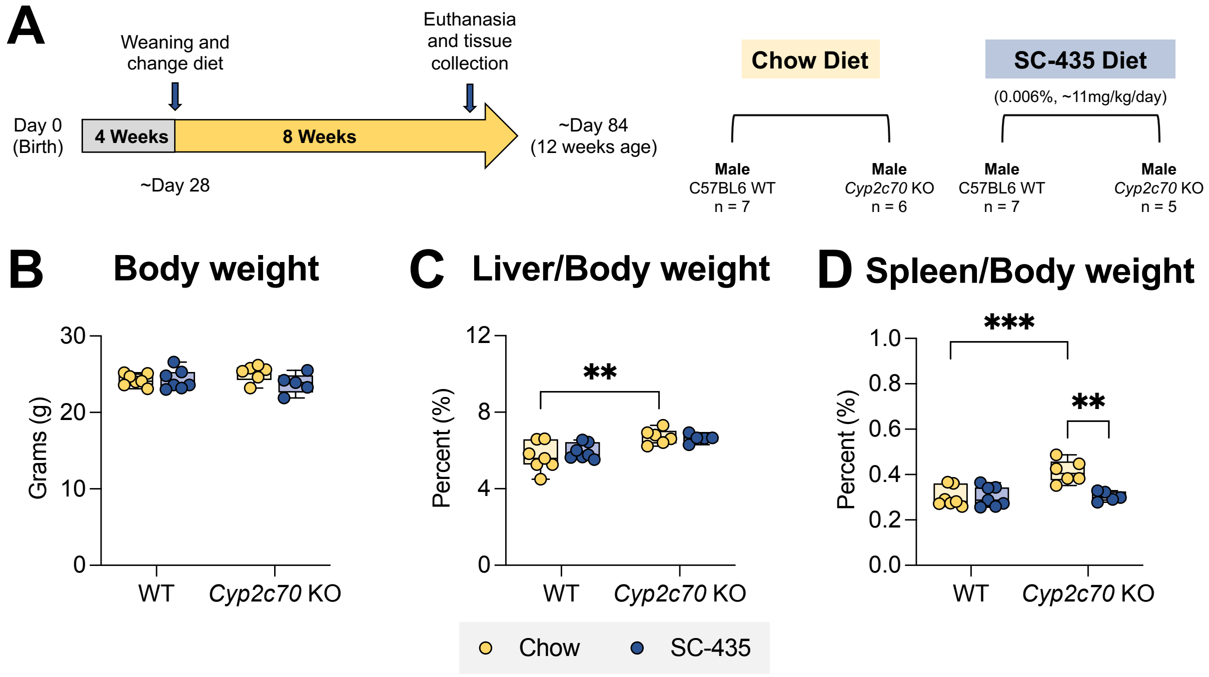

Fig. S2. Liver and spleen weights are elevated in male *Cyp2c70* KO mice. A: Experimental scheme. Mice are weaned at 4 weeks and maintained on chow or switched to SC-435 diet until sacrifice and tissue was collected at 12 weeks of age. B: Body weights. C: Liver/Body weights. D: Spleen/Body weights. Asterisks indicate significant differences between groups. Median values (line), interquartile range (boxes) and min to max values are shown (**$P < 0.01$, ***$P < 0.001$); n = 5 – 9 mice per group.

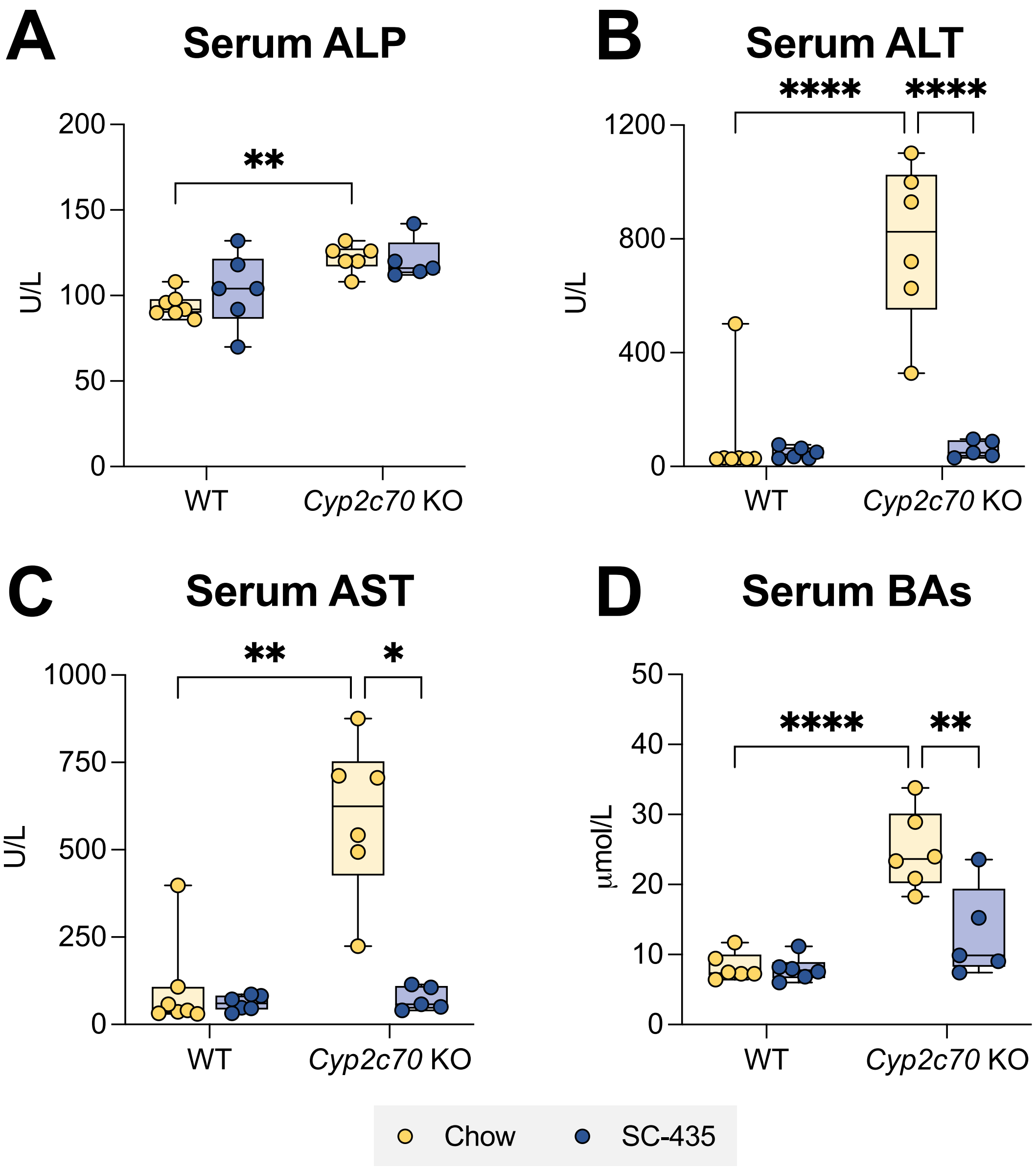

Fig. S3. Male *Cyp2c70* KO mice have elevated serum liver injury markers and are protected from cholestatic injury by the IBAT inhibitor SC-435. A: Serum alkaline phosphatase (ALP). B: Serum alanine aminotransferase (ALT). C: Serum aspartate aminotransferase (AST). D: Serum bile acids (BAs). Asterisks indicate significant differences between groups. Median values (*line*), interquartile range (*boxes*) and min to max values are shown (*whiskers*) (*$P < 0.05$, **$P < 0.01$, ****$P < 0.0001$); n = 5 – 9 mice per group.

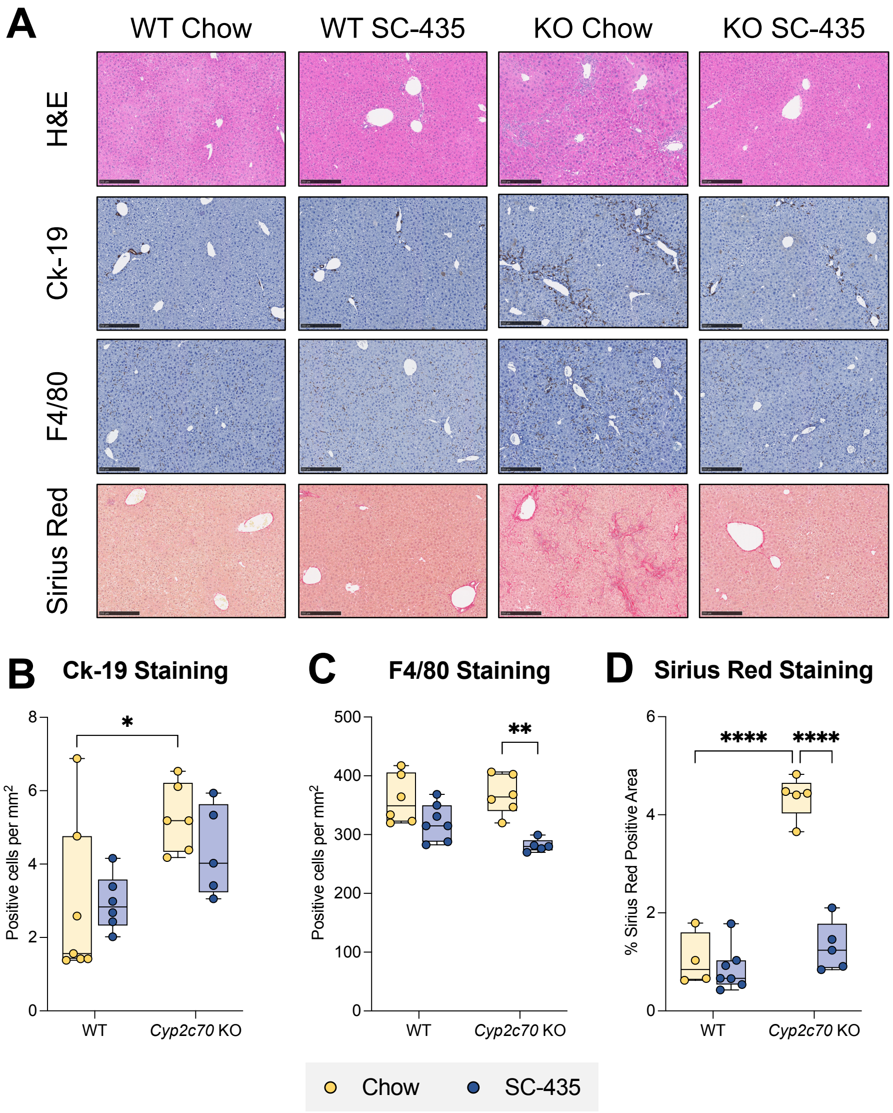

Fig. S4. Immune and fibrotic responses in male *Cyp2c70* KO mice are alleviated with SC-435 treatment. A: Morphological response to SC-435 treatment in WT and *Cyp2c70* KO mice. From top to bottom panel: Hematoxylin and eosin (H&E), Cytokeratin-19 (Ck-19), F4/80, and Sirius Red stained liver sections (original magnification 10X) from the indicated genotypes and treatments groups. *Scale bar*, 250 µm. Quantification of positive B: Ck-19, C: F4/80 cells per mm^2, and D: Percent Sirius Red positive area of the entire liver section. Asterisks indicate significant differences between groups. Median values (*line*), interquartile range (*boxes*) and min to max values are shown (*whiskers*) (*$P < 0.05$, **$P < 0.01$, ****$P < 0.0001$); n = 5 – 9 mice per group.

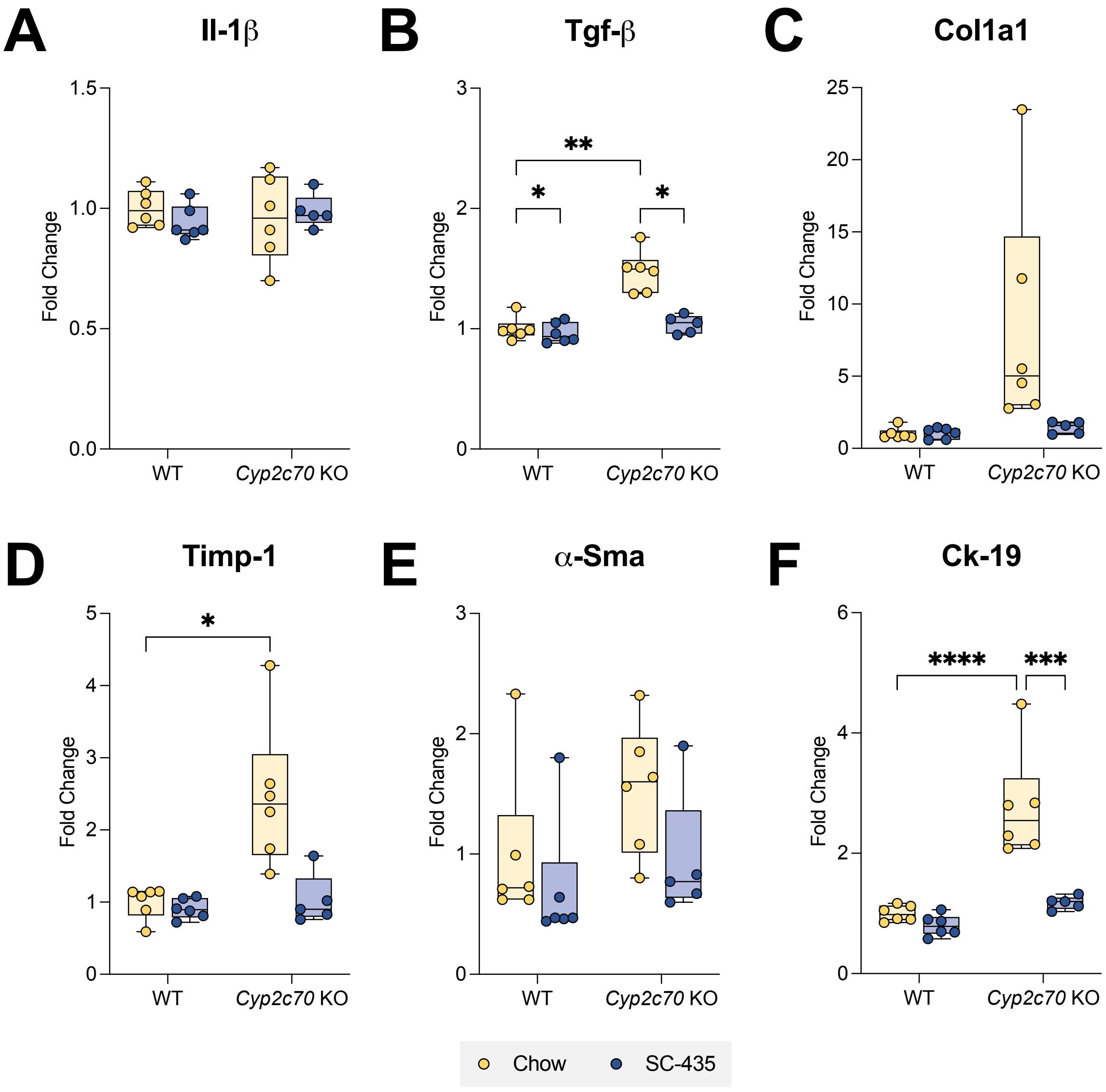

Fig. S5. Increased hepatic expression of inflammation and fibrosis-related genes in male *Cyp2c70* KO mice is alleviated with SC-435 treatment. A: Il-1β, B: Tgf-β, C: Col1a1, D: Timp-1, E: α-Sma, and F: Ck-19 gene expression. RNA was isolated from livers of individual mice and used for real-time PCR analysis. The mRNA expression was normalized using cyclophilin and the results for each gene are expressed relative to chow-fed WT mice for each gene. Asterisks indicate significant differences between groups. Median values (*line*), interquartile range (*boxes*) and min to max values are shown (*whiskers*) (**P*< 0.05, ***P* < 0.01, ****P* < 0.001; *****P* < 0.0001); n = 5 – 9 mice per group.

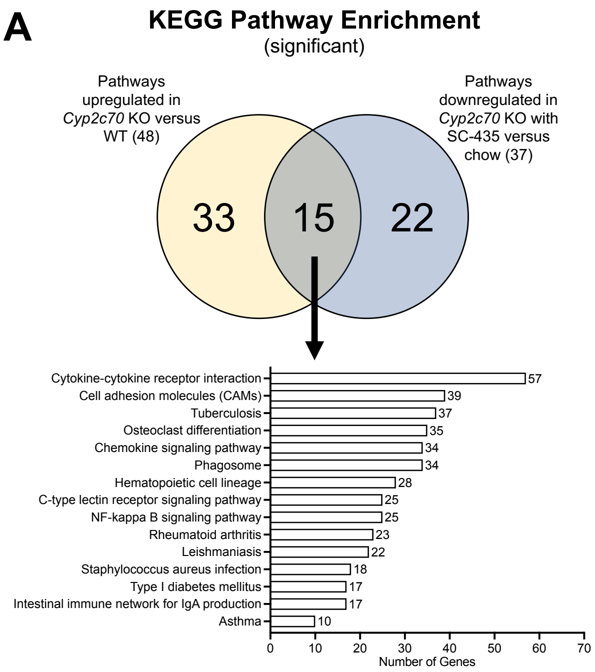

Fig. S6. KEGG enrichment analysis of significant pathways modified by IBAT inhibition in female *Cyp2c70* KO mice. A: Common KEGG pathways repressed by IBAT inhibition that are elevated in absence of *Cyp2c70*. B: Common KEGG pathways activated by IBAT inhibition that are suppressed in absence of *Cyp2c70*.

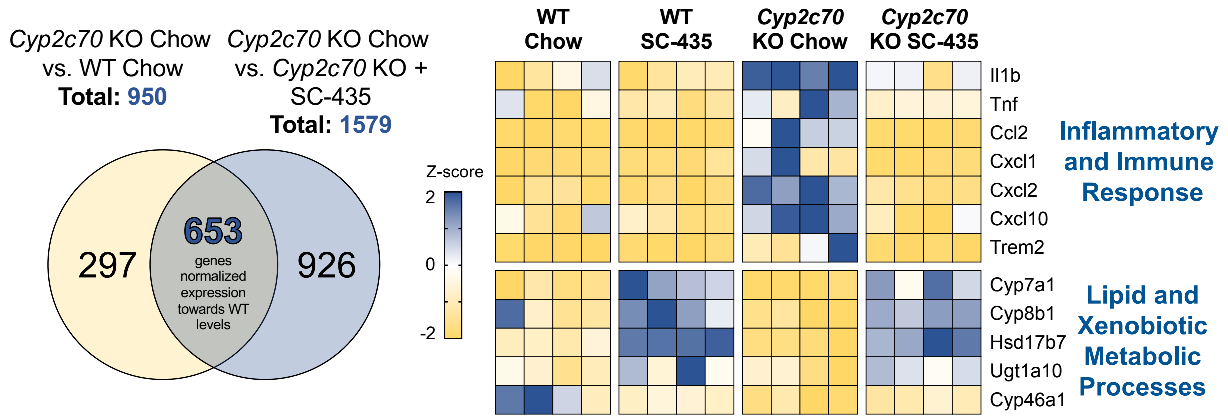

Fig. S7. Male mouse liver RNA-Seq analysis. Venn diagram showing the number of differentially expressed genes identified in *Cyp2c70 KO* mice versus WT, and *Cyp2c70* KO mice fed chow versus chow plus SC-435. Intersection represents the subset of genes whose expression is normalized towards WT levels. Change in expression are displayed as change in Z-score on heatmap for selected representative genes from indicated the pathways. Each column represents an individual animal.

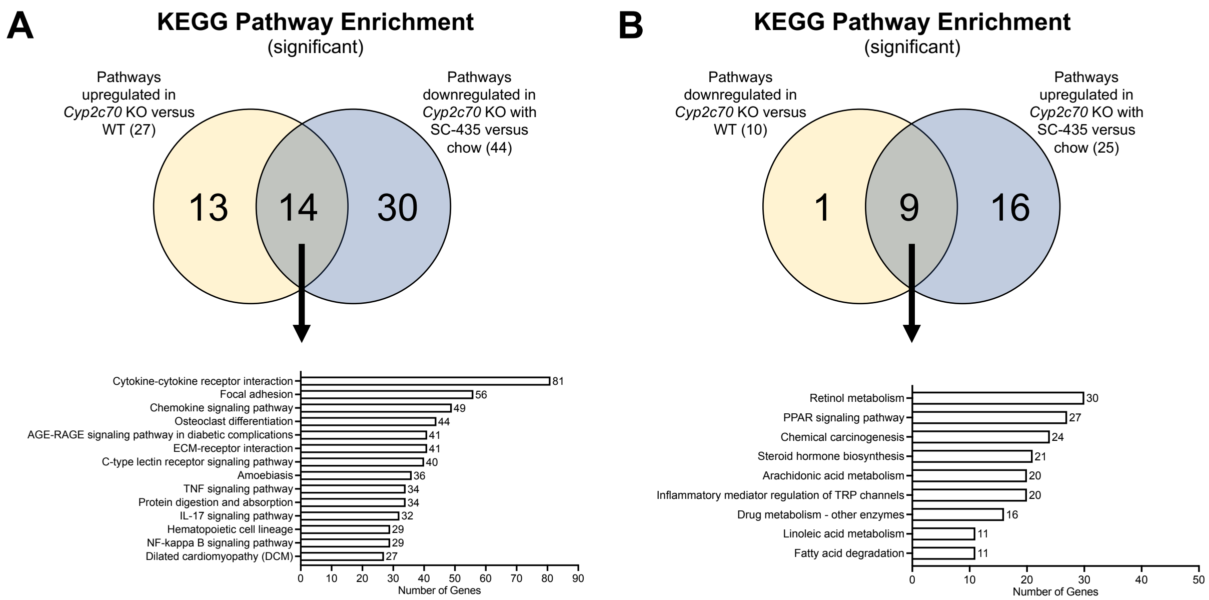

Fig. S8. KEGG enrichment analysis of significant pathways modified by IBAT inhibition in male *Cyp2c70* KO mice. A: Common KEGG pathways repressed by IBAT inhibition that are elevated in absence of *Cyp2c70*. B: Common KEGG pathways activated by IBAT inhibition that are suppressed in absence of *Cyp2c70*.

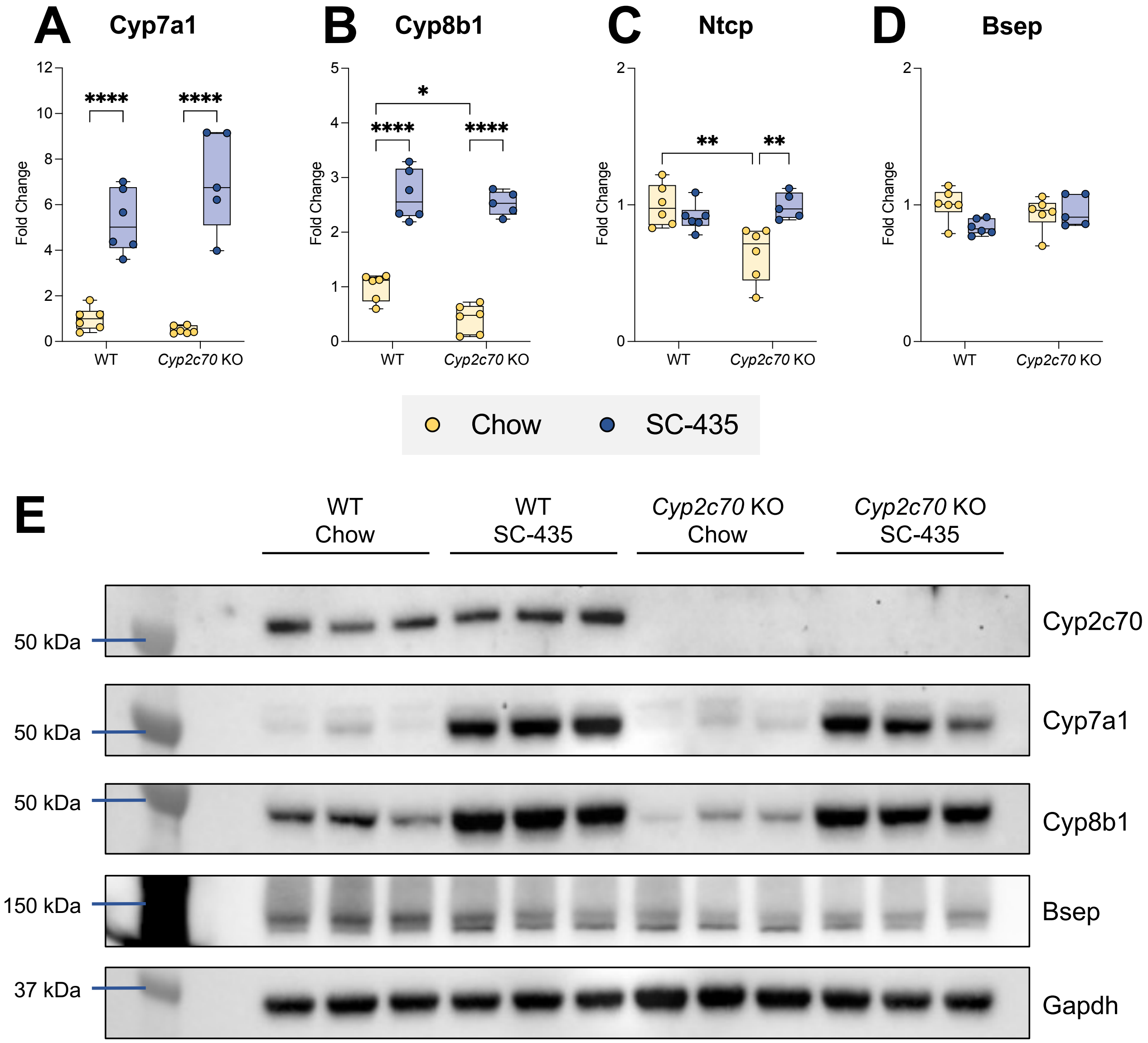

Fig. S9. IBAT inhibition increases hepatic expression of BA metabolism genes in male WT and *Cyp2c70* KO mice. RNA was isolated from livers of individual mice and used for real-time PCR analysis. The mRNA expression was normalized using cyclophilin and the results for each gene are expressed relative to chow-fed WT mice for each gene. A: Cyp7a1; B: Cyp8b1; C: Ntcp; D: Bsep. Asterisks indicate significant differences between groups. Median values (*line*), interquartile range (*boxes*) and min to max values are shown (*whiskers*) (*$P < 0.05$, **$P < 0.01$, ****$P < 0.0001$); n = 5 – 9 mice per group. E: Protein was isolated from livers of individual mice and used for Western Blot analysis, n = 3 mice per group.

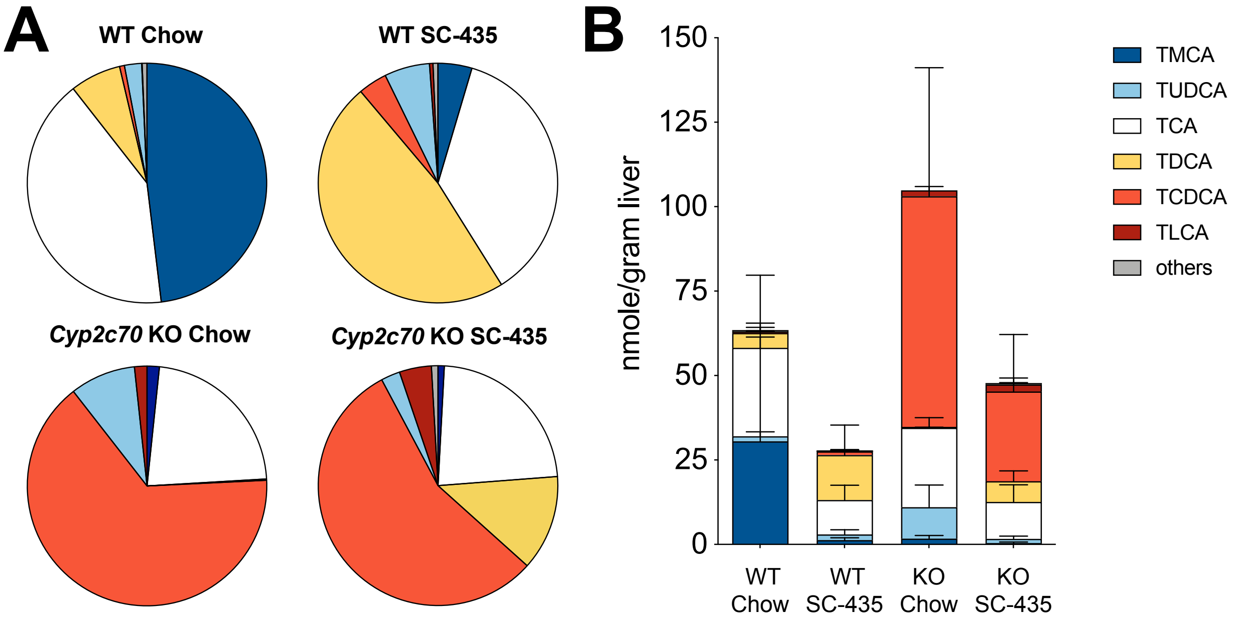

Fig. S10. IBAT inhibition prevents the increase in liver BA retention in male *Cyp2c70* KO mice. A: Muricholates are absent in *Cyp2c70* KO mice. Pie charts for the liver BA profiles. B: Absence of *Cyp2c70* increased the total amount of liver BAs and the amount of taurochenodeoxycholic acid (TCDCA) in *Cyp2c70* KO mice. Mean values $\pm$ SD are shown; n = 4 mice per group.

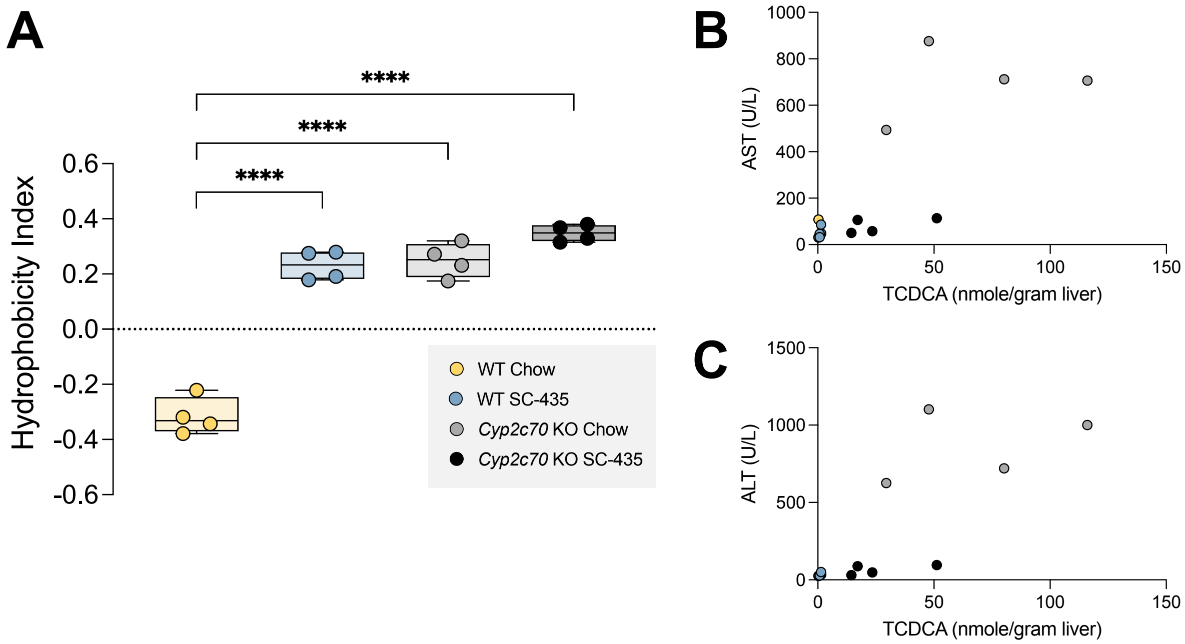

Fig. S11. IBAT inhibition does not reduce liver BA hydrophobicity in male *Cyp2c70* KO mice. A: Liver BA composition was used to calculate the hydrophobicity index. IBAT inhibition and inactivation of *Cyp2c70* increases the hydrophobicity of the liver BA pool. Asterisks indicate significant differences between groups. Median values (*line*), interquartile range (*boxes*) and min to max values are shown (*whiskers*) (*****P* < 0.001); n = 4 mice per group. B and C: Total liver taurochenodeoxycholic acid (TCDCA) and serum levels of alanine aminotransferase (ALT) and aspartate aminotransferase (AST) are reduced in IBAT inhibitor treated *Cyp2c70* KO mice.
